# Supplementary material for: Defective mitochondria remodelling in B cells leads to an aged immune response
Source: Nat Commun. 2024 Mar 22;15:2569. doi: 10.1038/s41467-024-46763-1 (PMC10960012; doi:10.1038/s41467-024-46763-1)
Supplement: Supplementary file 1 — Supplementary Information [file 41467_2024_46763_MOESM1_ESM.pdf]

Supplementary Information

Defective mitochondria remodelling in B cells leads to an aged immune response

Marta Iborra-Pernichi; Nuria Martínez-Martín*

*For correspondence: nmartinez@cbm.csic.es

Supplementary figures

Suppl Fig 1

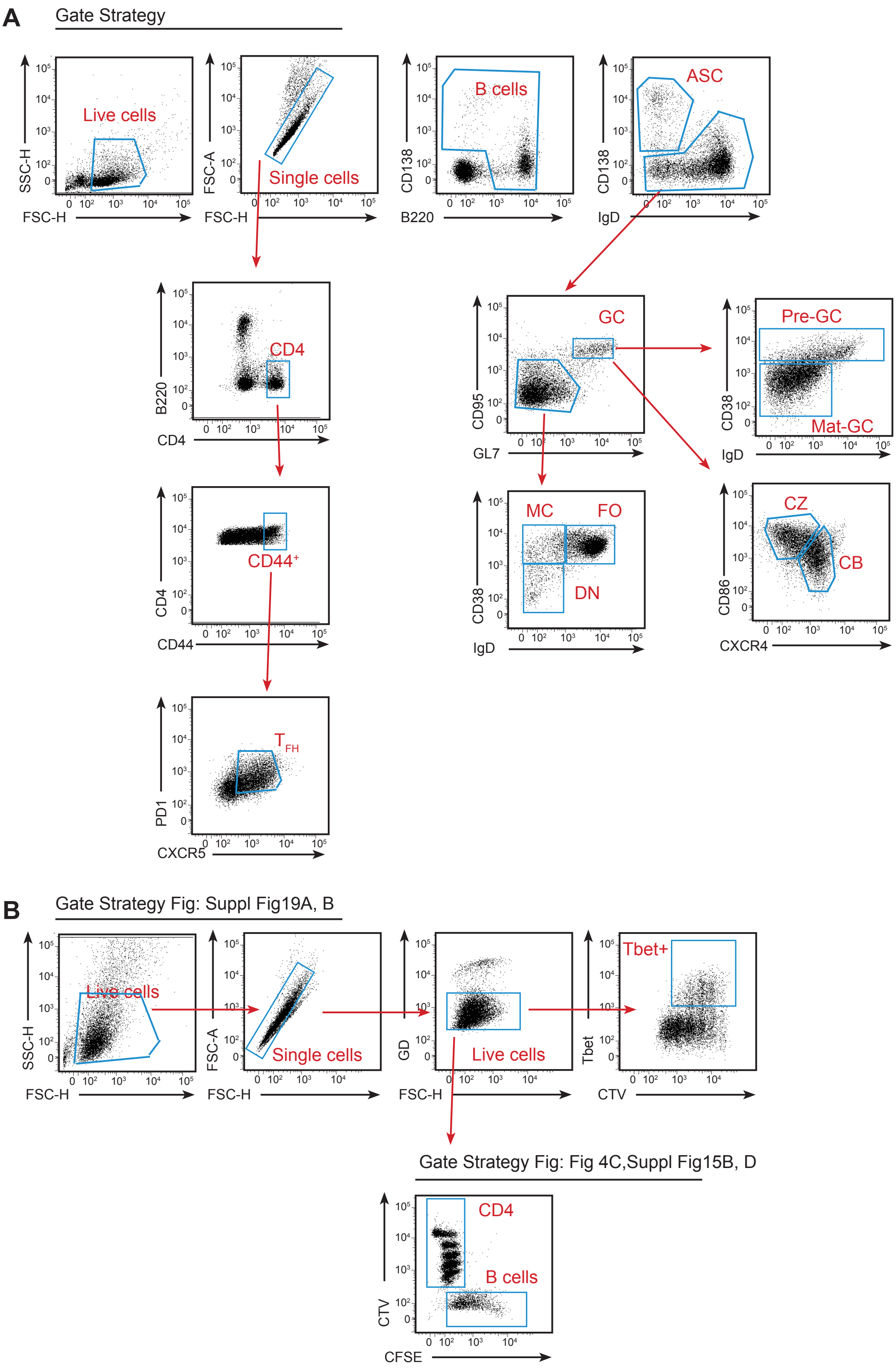

Suppl. Fig 1.- Gating strategies used for cell population charcaterization by Flow Citometry and cell sorting

(A) Main B and T cell population after immunization

(B) B and T cell population after in vitro culture

Suppl Fig 2

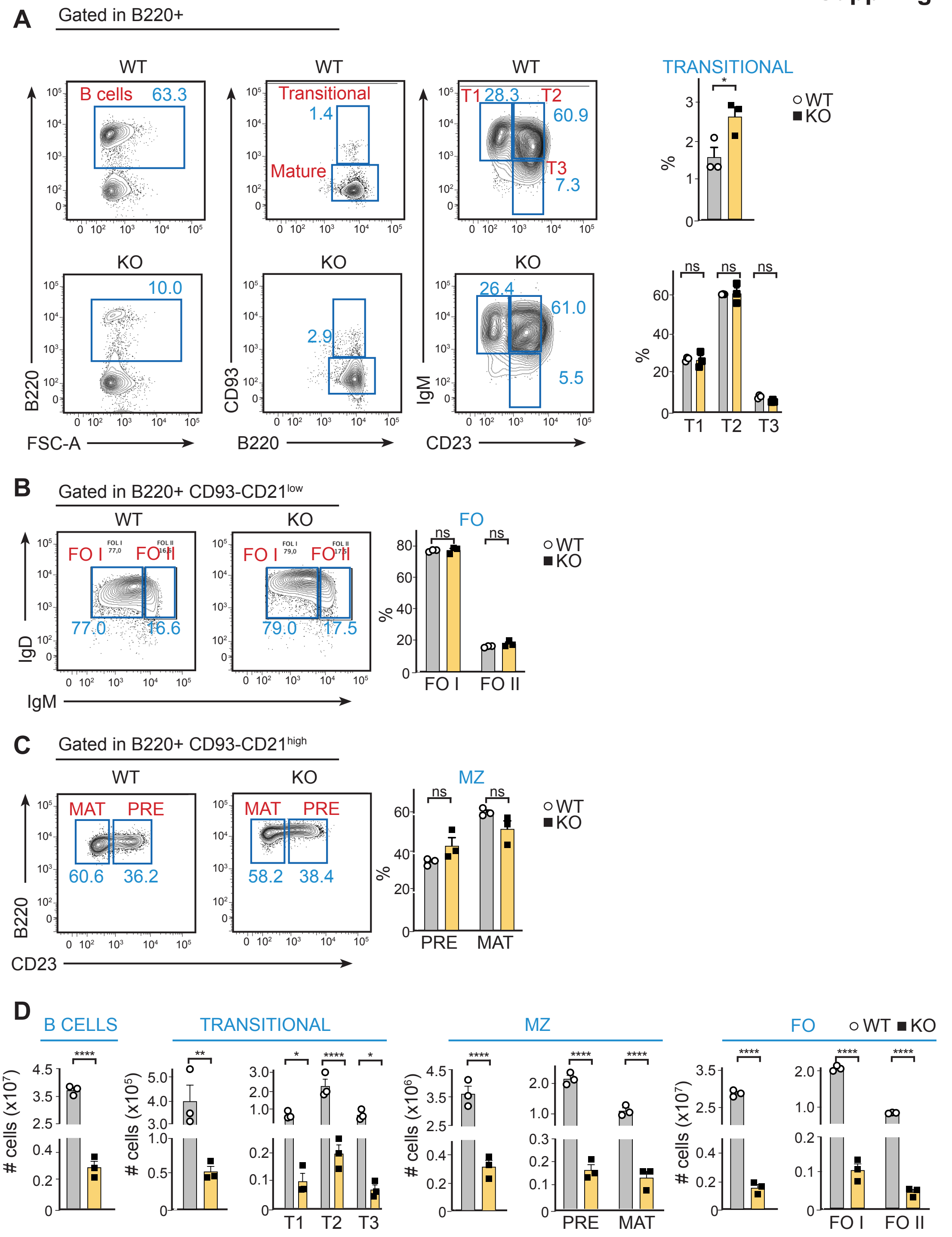

A
Gated in B220+
WT
KO
B cells
63.3
10.0
Transitional
1.4
Mature
2.9
T1
28.3
T2
60.9
T3
7.3
26.4
61.0
5.5
B220
FSC-A
CD93
IgM
CD23
TRANSITIONAL
%
T1
T2
T3
ns
WT
KO
B
Gated in B220+ CD93-CD21low
FO I
FO II
77.0
16.6
79.0
17.5
IgD
IgM
FO
C
Gated in B220+ CD93-CD21high
MAT
PRE
60.6
36.2
58.2
38.4
MZ
D
B CELLS
TRANSITIONAL
MZ
FO
cells (x10^7)
cells (x10^5)
cells (x10^6)

Suppl. Fig. 2.- *Tfam* deletion in B cells reduces mature B cell output from the bone marrow.

(A) Flow cytometry plots for splenic B cells showing mature ($B220^+$, $CD93^-$) and transitional B cell population ($B220^+$, $CD93^+$) in non-immunised WT and *Tfam* KO mice. IgM and CD23 distribution illustrates the maturation of Transitional B cells. Bar charts indicate the percentage of B cell populations gated in plots**.**

(B-C) Splenic Mature B cell compartment in non-immunize WT and *Tfam* KO mice. Flow cytometry plot of Follicular B cells (FO) gated as $B220^+$, $CD93^-$, $CD21^{lo}$ (B). IgD and IgM distribution illustrates FO maturation as FO type I (FO I) and FO type II (FO II). Flow cytometry plot of Marginal Zone (MZ) B cells gated as $B220^+$, $CD93^-$, $CD21^{high}$ (C). CD23 distribution illustrates the maturation state of MZ cells, Pre MZ ($CD23^+$) and mature (MAT) MZ ($CD23^-$). Bar chart indicates the percentage of B cell populations.

(D) Absolut count of B cell populations gated on panels A-C.

In all panels, bar charts show the quantification of one representative experiment out of three, and error bars represent mean +- SEM. Each dot represents one mouse. In panel A upper bar chart, and panel D unpaired two-tail t-test was conducted. For rest of panels, Two-way ANOVA was conducted with Tukey's multiple comparisons test. P vaules: * $p<0.05$, ** $p<0.01$, ***$p<0.001$ and ****$p<0.0001$. Source data are provided as a Source Data file.

Suppl Fig 3

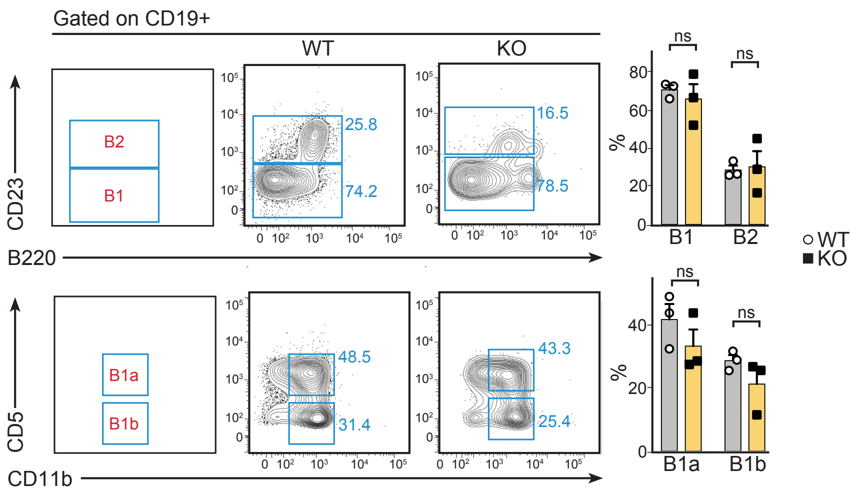

Suppl. Fig. 3.- *Tfam* deletion in B cells does not impact the distribution of B cell populations in the peritoneum.

Upper panel: Gating strategy and flow cytometry plots for B cells from the peritoneal wash (gated on CD19^{+}) according to the expression of CD23 (B1 as CD23^{-} and B2 as CD23^{+}). Lower panel: gating strategy and flow cytometry plots for B1 characterisation based on CD11b and CD5 (B1a CD11b^{+} CD5^{+} and B2 CD11b^{+} CD5^{-}). Bar charts indicate the percentage of each B cell population of non-immunised WT and *Tfam* KO mice.

In all panels, bar charts show the quantification of one representative experiment out of three, and error bars represent mean +- SEM. Each dot represents one mouse Two-way ANOVA was conducted with Tukey's multiple comparisons test. P-values: * p<0.05, ** p<0.01, ***p<0.001, and ****p<0.0001. Source data are provided as a Source Data file.

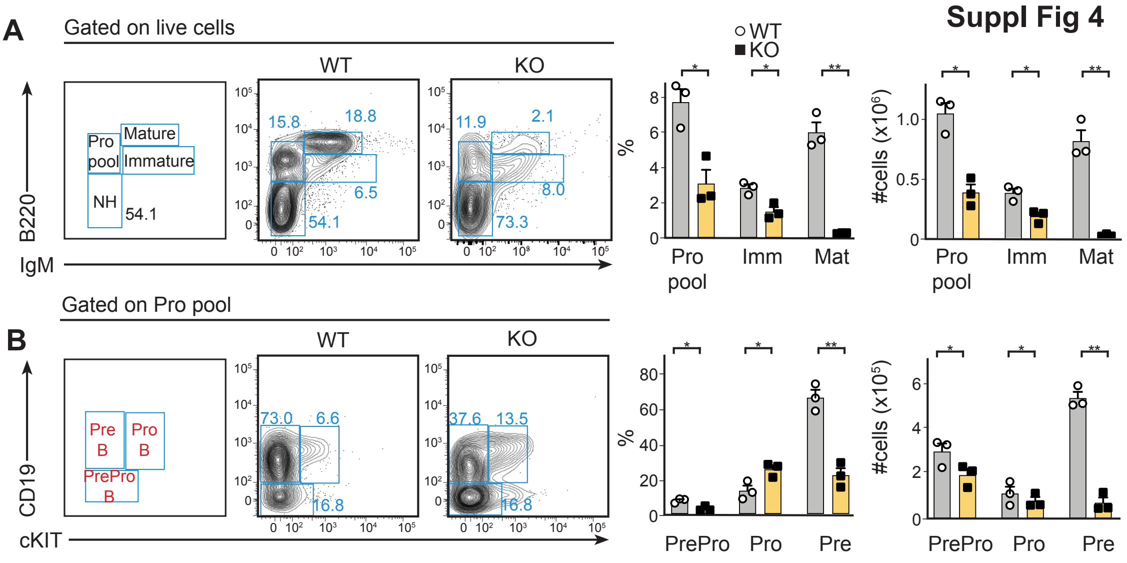

Suppl. Fig. 4.- *Tfam* deletion in B cells leads to an early B cell development blockage in the bone marrow.

(A) Gating strategy and flow cytometry plots for bone marrow B cell subsets (gated on single cells) according to the expression of IgM and B220 defining the pro-pool (B220^{+} IgM^{-}), immature (B200^{+}, IgM^{+}) and mature (B220^{hi} IgM^{+}) B cell populations.

(B) Gating strategy for pro-pool B cells based on the expression of CD19 and CD117 (cKit) defining pre-pro B (Ckit^{-} CD19^{-}), pro-B (Ckit^{+} CD19^{+}), Pre B (Ckit-CD19^{+}) cell subsets. Bar charts indicate the percentage of each B cell population in non-immunised WT and *Tfam* KO mice.

In all panels, bar charts show the quantification of one representative experiment out of three, and error bars represent mean +- SEM. Each dot represents one mouse. Two-way ANOVA was conducted with Tukey's multiple comparisons test: * p<0.05 and ** p<0.01.0001. Source data are provided as a Source Data file.

Suppl Fig 5

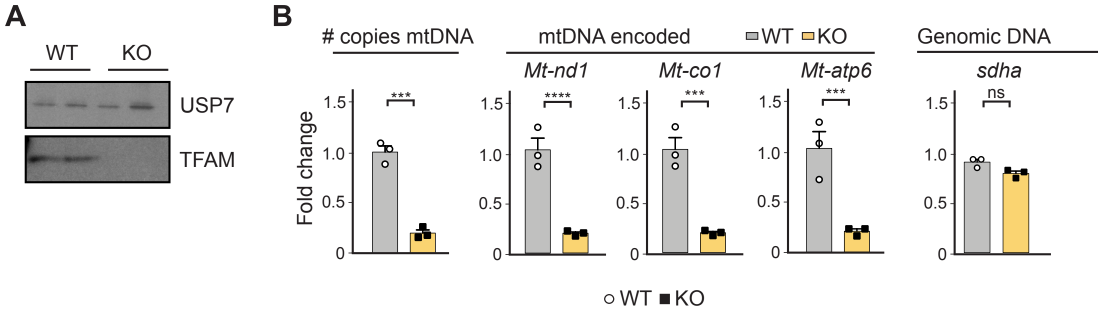

Suppl. Fig. 5.- *Tfam* controls mt-DNA copy numbers and is required to transcribe mt-encoded genes in B cells.

(A) Immunoblot of TFAM and USP7 (loading control) levels in WT and *Tfam* KO purified B cells.

(B) Left panel, quantification of copy number of mitochondrial DNA (mtDNA) quantified according to a comparison of *Mt-nd1* expression relative to Hexokinase 2 (*Hk2*) DNA expression. Expression of mt-encoded genes *nd1*, *co1* and *atp6* was determined by RT-qPCR. Right, Genomic DNA quantification was determined by levels of nuclear-encoded *Sdha*

In all panels, bar charts show the quantification of one representative experiment out of three, and error bars represent mean +- SEM. Each dot represents one mouse. Unpaired two-tail t-test : ***p<0.001, and ****p<0.0001. Source data are provided as a Source Data file.

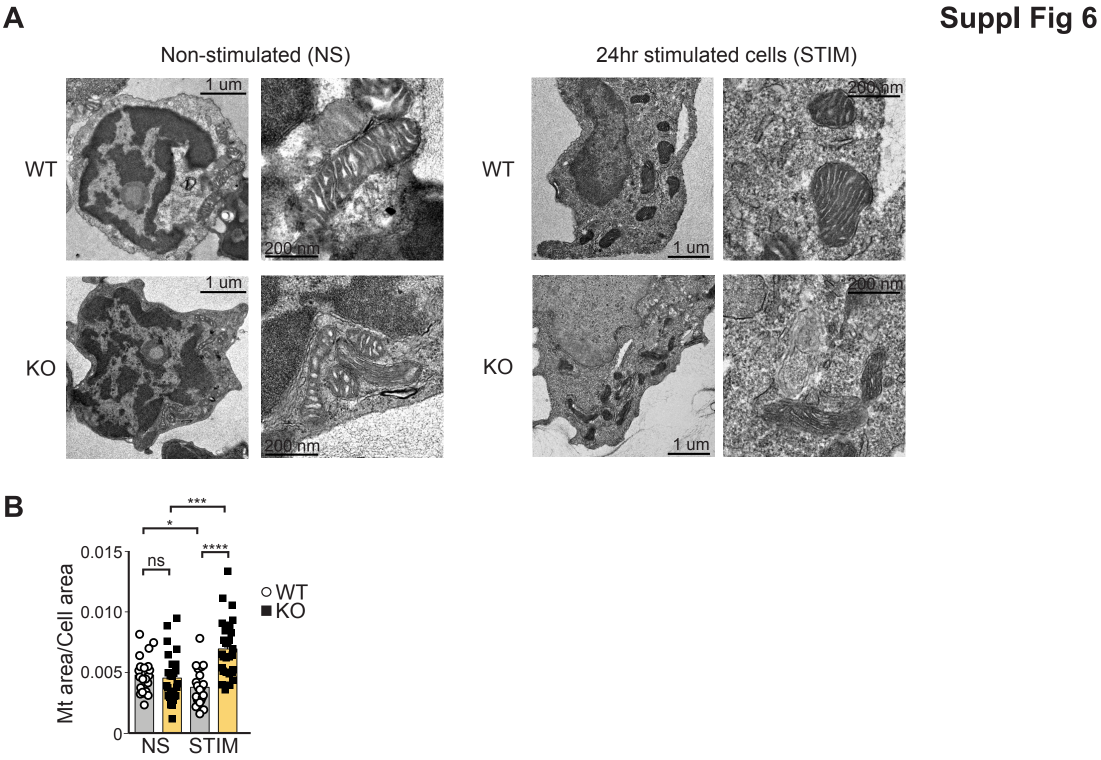

Suppl. Fig. 6.- *Tfam* is required for mitochondria remodelling upon antigen encounter.

(A) Representative electron microscopy field of WT and *Tfam* KO cells pre- (NS) and post-stimulation (STIM) with anti-CD40 and anti-IgM.

(B) Images were analysed using Image J software, and the total mitochondrial area was quantified and normalised over the cell area.

Bar charts show the quantification of one representative experiment out of three; error bars represent mean +- SEM. Each dot represents one cell. Two-way ANOVA was conducted with Tukey's multiple comparisons test: * $p<0.05$, ***$p<0.001$, and ****$p<0.0001$. Source data are provided as a Source Data file.

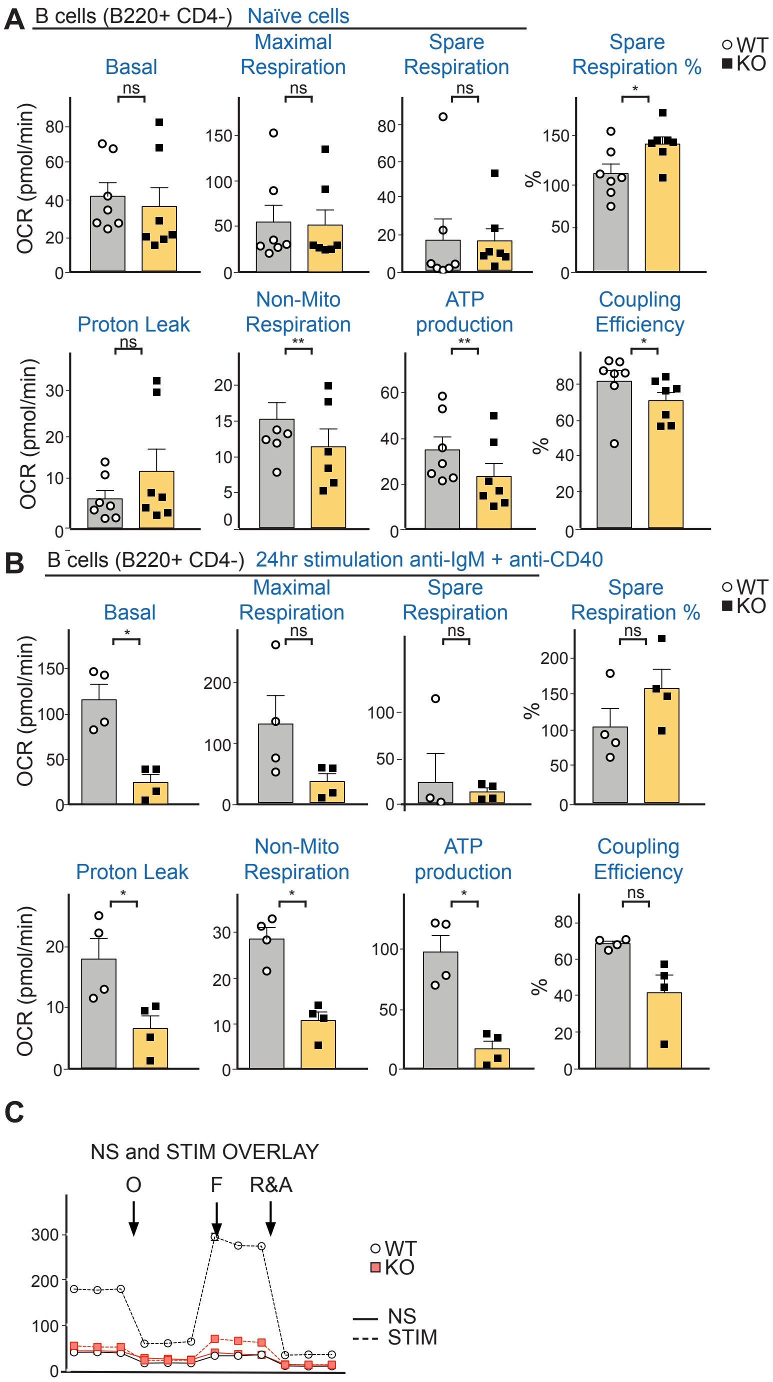

Suppl. Fig. 7.- *Tfam* drives mitochondrial remodelling in B cells upon antigen encounter.

(A-B) Bar charts showing oxygen consumption rates (OCR) values of Basal, Maximal, Spare respiration, Proton leak, Non-mitochondria respiration, and ATP production of non-stimulated (A) and 24h-stimulated (B) B cells. Right bar charts indicate the percentage of spare respiration and coupling efficiency.

(C) Representative graph of extracellular flux analysis of non-stimulated (NS) and stimulated (STIM) purified B cells from WT and *Tfam* KO mice. Basal OCR was measured before and after injection of oligomycin (O), FCCP (F) and a combination of rotenone plus antimycin A (R&A).

In all panels, bar charts show the quantification of one representative experiment out of three, and error bars represent mean +- SEM. Each dot represents one mouse. Unpaired two-tail t-test: *$p<0.05$, and **$p<0.01$. Source data are provided as a Source Data file.

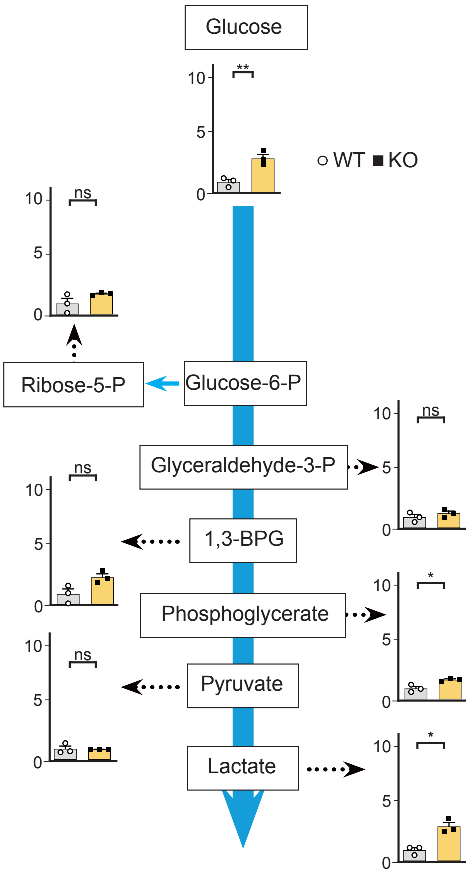

Suppl. Fig. 8.- Metabolomic analysis shows increased levels of glycolytic intermediates in *Tfam* KO B cells.

Bar chart indicating the relative amount of compounds related to glucose metabolism in cell lysates from WT and *Tfam* KO B cells after 24h-stimulation.

Each dot represents one mouse and is the average of 3 technical replicates. Error bars represent mean +- SEM. Unpaired two-tail t-test: * p<0.05, and ** p<0.01. Source data are provided as a Source Data file.

Suppl Fig 9

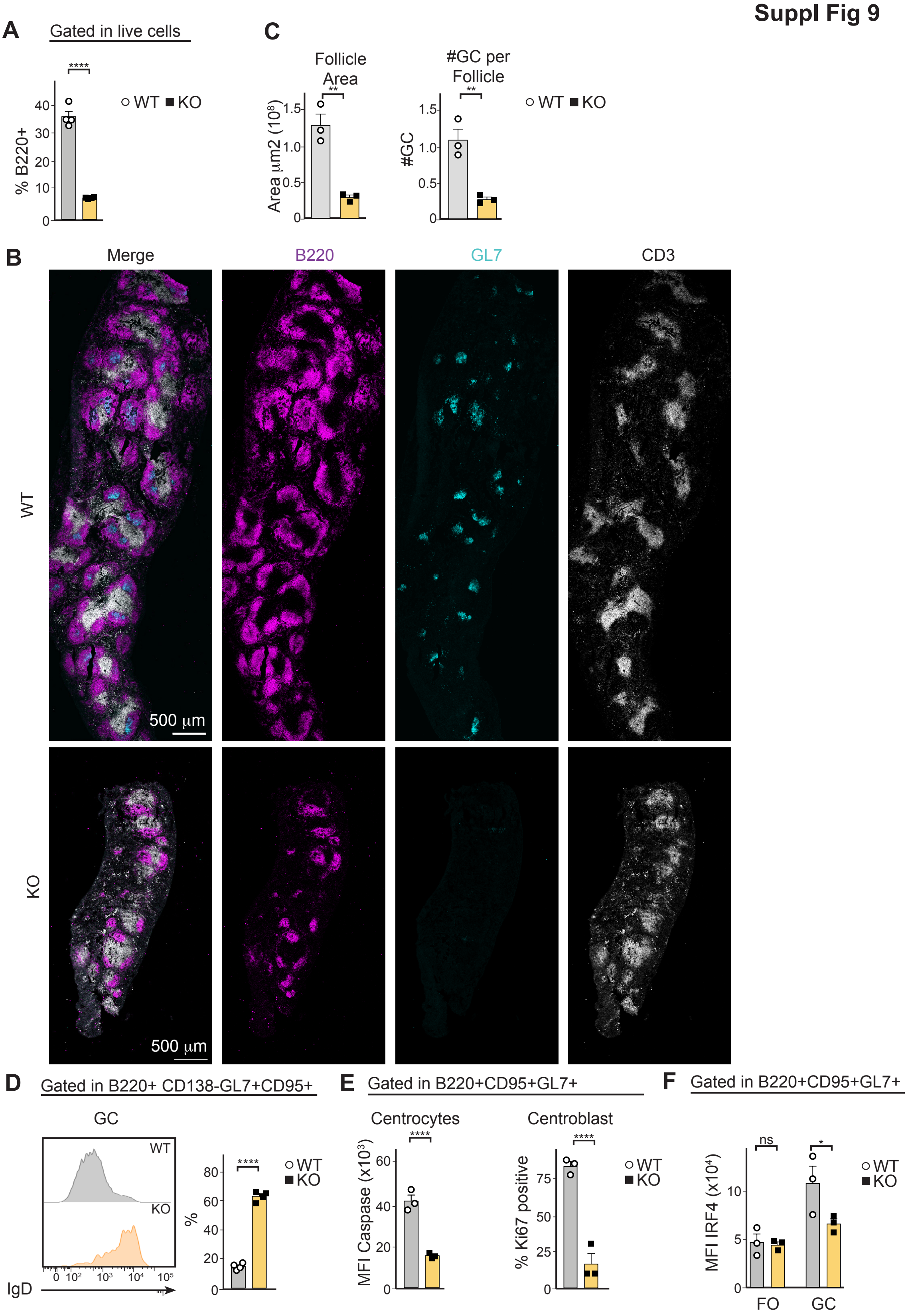
A
Gated in live cells
% B220+
WT KO
****
C
Follicle Area
Area µm2 (10⁸)
#GC per Follicle
#GC
**
B
Merge
B220
GL7
CD3
WT
KO
500 µm
D
Gated in B220+ CD138-GL7+CD95+
GC
IgD
%
****
E
Gated in B220+CD95+GL7+
Centrocytes
Centroblast
MFI Caspase (x10³)
% Ki67 positive
F
Gated in B220+CD95+GL7+
MFI IRF4 (x10⁴)
ns
*
FO
GC

Suppl. Fig. 9.-*Tfam* deletion in B cells leads to a blockage in GC reaction

(A) Bar chart indicating the proportion of B cells ($B220^+$) in WT and *Tfam* KO after immunisation (Sheep red blood cells (SRBC); 7 days post immunisation (p.i)).

(B-C) Confocal microscopy (5x objective) tile images of the spleen of immunised WT and *Tfam* KO mice show B220, GL7, and CD3 staining (500mm). (C) Bar chart indicates the area of $B220^+$ (Follicle area) in μm2 and the quantity of GC per follicle. Images were quantified using Fiji.

(D) Representative flow cytometry histograms and quantification of IgD levels in GC ($B220^+CD138^-GL7^+CD95^+$) B cells of WT and *Tfam* KO mice 7 days p.i.

(E) Left, bar chart indicating cell death in centrocyte cell population: Cell death is represented by caspase protein levels. Right, a bar chart showing the proportion of proliferative centroblasts as Ki67+ cells.

(F) Bar chart indicating levels of IRF4 protein measured by FACS in Follicular (FO) and Germinal centre (GC) cells from WT and KO mice 7 days p.i.

In all panels, bar charts show the quantification of one representative experiment out of three, and error bars represent mean +- SEM. Each dot represents one mouse. Unpaired two-tailed t-test: *P<0,05, ** p<0,01, ***p<0.001 and ****p<0.0001. Source data are provided as a Source Data file.

Suppl Fig 10

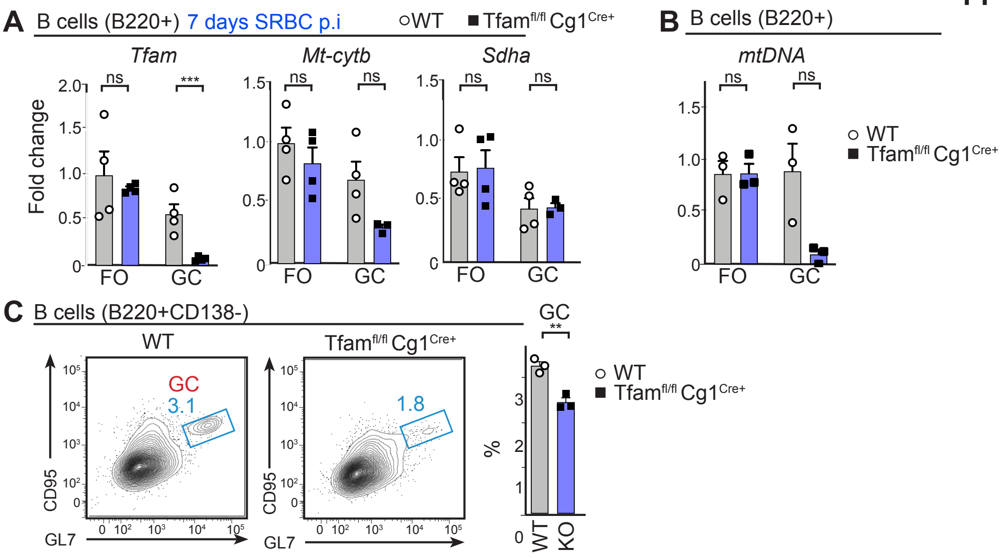

Suppl. Fig 10.- Specific deletion of TFAM in activated B cells leads to a blockage of the GC reaction.

(A) From left to right, relative mRNA levels of *Tfam*, *Mt-cyb* and *Sdha* determined by RT-PCR in sorted Follicular cells (FO) and Germinal Centre (GC) B cells of immunised WT and *Tfam*$^{f/f}$Cγ1^{Cre+} mice. Bar chart indicates fold change from FO WT.

(B) Quantification of the mitochondrial DNA copy number (mtDNA) quantified according to a comparison of *Atp6* expression relative to Hexokinase 2 (*Hk2*) DNA expression in sorted FO and GC cells from immunised WT and *Tfam*$^{f/f}$Cγ1^{Cre+}.

(C) Left, flow cytometry plots showing splenic germinal centre (GC, CD95$^+$, GL7$^+$) and FO (CD95$^-$, GL7$^-$) B cell populations 7 days post immunisation (p.i) in WT and *Tfam*$^{f/f}$Cγ1^{Cre+} mice. Right, corresponding bar charts indicate the proportion of GC B cells (gated in B220$^+$CD138$^-$).

In all panels, bar charts show the quantification of one representative experiment out of three, and error bars represent mean +- SEM. Each dot represents one mouse. Unpaired two-tailed t-test : ** p<0.01, and ***p<0.001. Source data are provided as a Source Data file.

Suppl Fig 11

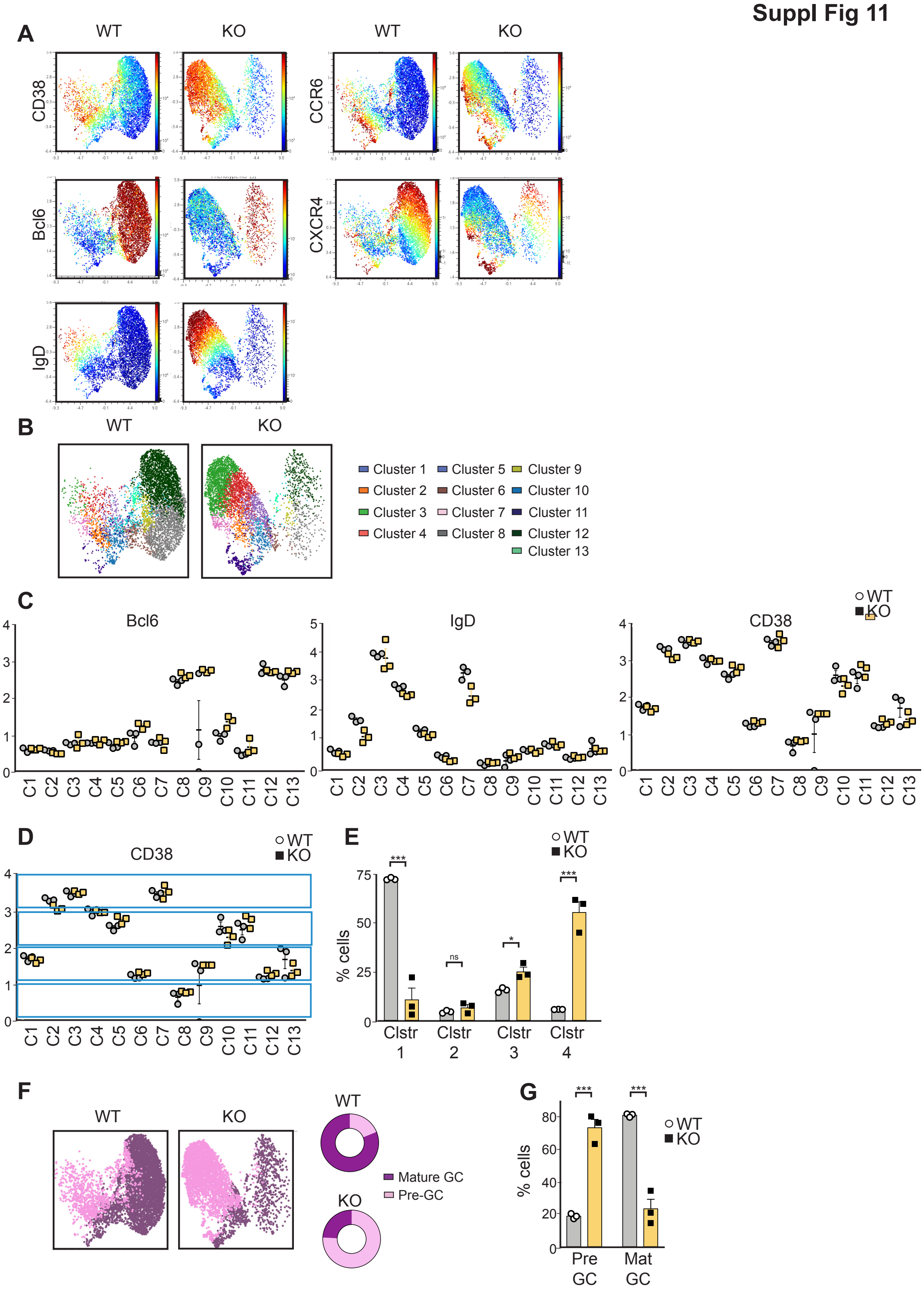
A
WT
KO
CD38
CCR6
Bcl6
CXCR4
IgD
B
Cluster 1
Cluster 2
Cluster 3
Cluster 4
Cluster 5
Cluster 6
Cluster 7
Cluster 8
Cluster 9
Cluster 10
Cluster 11
Cluster 12
Cluster 13
C
Bcl6
IgD
CD38
C1 C2 C3 C4 C5 C6 C7 C8 C9 C10 C11 C12 C13
D
CD38
E
% cells
Clstr 1
Clstr 2
Clstr 3
Clstr 4
***
ns
*
F
Mature GC
Pre-GC
G
Pre GC
Mat GC

Suppl. Fig. 11.- *Tfam* deficiency on B cells promotes a GC maturation blockage, as shown in Elbow FSOM analysis by OMIQ.

(A) UMAP distribution of cell markers used to define clusters in GC of WT and *Tfam* KO mice 7 days post immunisation (p.i) with SRBC.

(B) UMAP distribution of clusters generated by elbow FSOM analysis.

(C) Graphs indicating the relative amount of Bcl6, IgD and CD38 in clusters generated by elbow FSOM analysis.

(D) Graph representing the four areas obtained by subdividing the elbow FSOM-clusters using CD38 expression levels.

(E) Bar charts indicating the abundance of clusters generated by FSOM analysis.

(F-G) UMAP representation of pre-GC and mat-GC manually gated based on CD38, IgD. Analysis was performed manually in downsampling total GC B cells gated as $B220^+$ $GL7^+$ $CD95^+$. (G) Bar charts showing percentage of pre-GC and mat-GC manually gated based on CD38, IGD. Analysis was performed manually in downsampling total GC B cells gated as $B220^+$ $GL7^+$ $CD95^+$.

In all panels, bar charts show the quantification of one representative experiment out of three, and error bars represent mean +- SEM. Each dot represents one mouse. Two-way ANOVA was conducted with Tukey's multiple comparisons test: * $p<0.05$, and *** $p<0.001$. Source data are provided as a Source Data file.

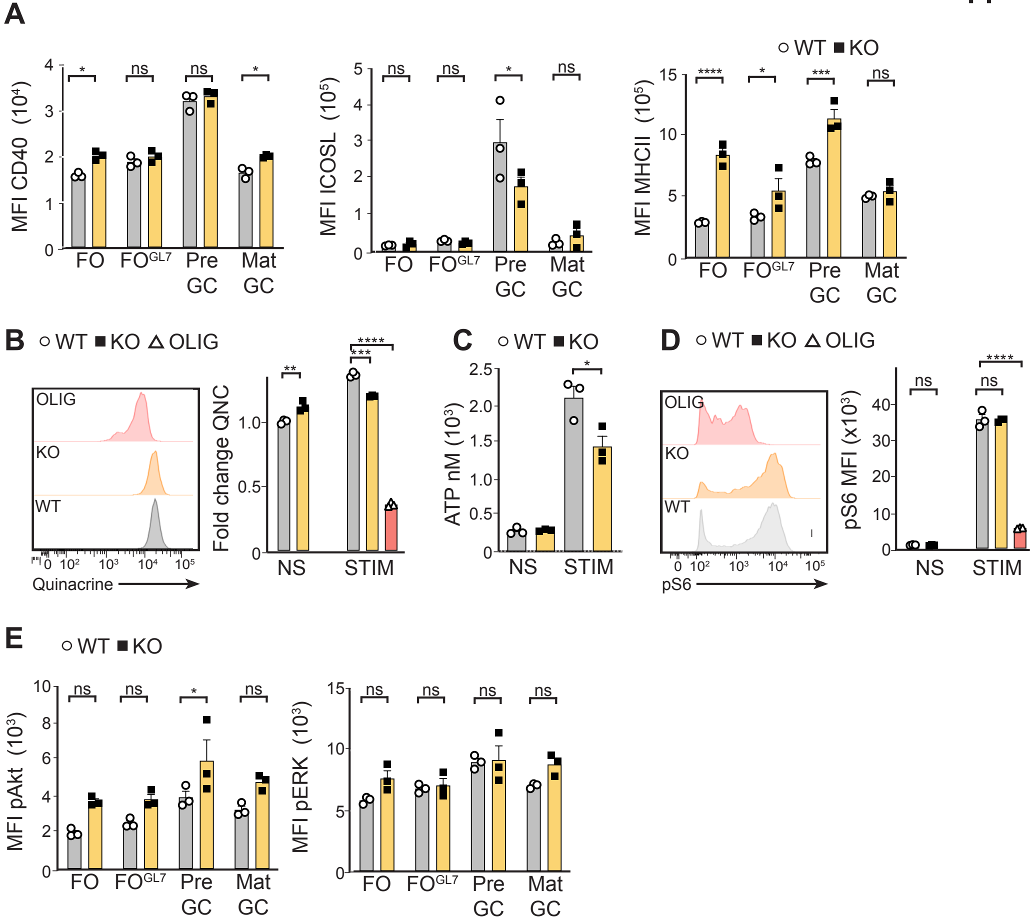

Suppl. Fig. 12.-. *Tfam* deletion unveils the B cell´s metabolic plasticity upon antigen encounter

(A) Quantification of MFI levels of CD40 (left), ICOSL (centre) and MHCII (right) during B cell activation in vivo (FO to mat-GC B cells) on WT and *Tfam* KO mice.

(B) Left, flow cytometry histograms showing MFI levels of quinacrine in WT and *Tfam* KO B cells. Oligomycin pre-treated B cells were used as a control of ATP drop. Quinacrine indicates relative ATP levels. Right, quantification of fold change increases in ATP levels in different conditions compared to non-stimulated (NS) WT B cells.

(C) The figure displays the relative ATP concentration (nM) in 24-hour cultured WT and *Tfam* KO purified B cells under non-stimulated conditions or stimulated with α-IgM + α-CD40. For measurement, 1.5x10^5 B cells were lysed in 100 µL Tris-EDTA buffer, and 10 µL of the lysate was utilized for luminescent assay (luciferase reaction).

(D) Left, flow cytometry analysis of pS6 levels in stimulated (STIM) cells and right, fold change increase quantification of pS6 levels in different conditions compared to NS WT.

(E) Quantification of pAKT (left) and pERK (right) levels during B cell differentiation (FO to mat-GC B cells) on WT and *Tfam* KO mice.

In all panels, bar charts show the quantification of one representative experiment out of three, and error bars represent mean +- SEM. Each dot represents one mouse. For panel A and E unpair two-tailed t-test was conducted, and for panel B, Cand D Two-way ANOVA was conducted with Tukey's multiple comparisons test: * $p<0.05$, ** $p<0.01$, ***$p<0.001$, and ****$p<0,0001$. Source data are provided as a Source Data file.

Suppl Fig 13

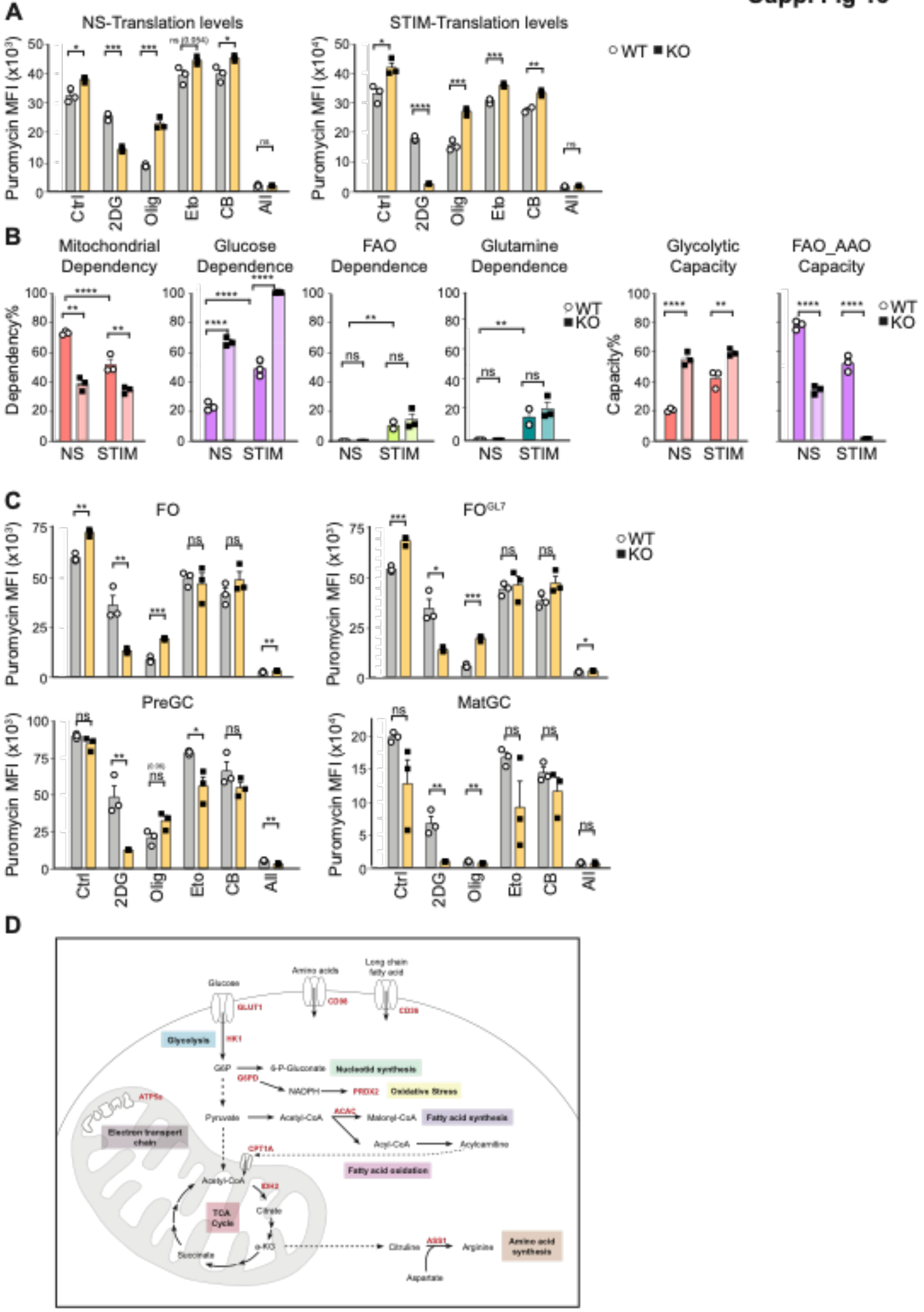
A
NS-Translation levels
STIM-Translation levels
Puromycin MFI (x10³)
Ctrl
2DG
Olig
Eto
CB
All
WT KO
B
Mitochondrial Dependency
Glucose Dependence
FAO Dependence
Glutamine Dependence
Glycolytic Capacity
FAO_AAO Capacity
Dependency%
Capacity%
NS
STIM
C
FO
FO^GL7
PreGC
MatGC
D
Glucose
Amino acids
Long chain fatty acid
GLUT1
CD98
CD36
Glycolysis
HK1
G6P
6-P-Gluconate
Nucleotid synthesis
G6PD
NADPH
PRDX2
Oxidative Stress
ATP5a
Electron transport chain
Pyruvate
Acetyl-CoA
ACAC
Malonyl-CoA
Fatty acid synthesis
Acyl-CoA
Acylcarnitine
CPT1A
Fatty acid oxidation
IDH2
Citrate
TCA Cycle
Succinate
α-KG
Citruline
ASS1
Arginine
Amino acid synthesis
Aspartate

Suppl. Fig. 13- *Tfam* deletion in B cells triggers metabolic plasticity to maintain bioenergetics homeostasis.

(A) Translation levels according to MFI of incorporated puromycin in purified NS (left) and STIM (right) B cells. Cells were incubated with vehicle (Ctrl), 2-Deoxi-D-glucose (2DG), Oligomycin (Olig), Etomoxir (Eto), and CB-839 (CB). A combination of all inhibitors (All) was used as a negative control for translation.

(B) Metabolic profile of B cell populations obtained by SCENITH as result of incubation with different inhibitors (Oligomycin gives Mitochondrial dependency, 2-deoxy-D-glucose (2DG) for glucose dependency, Etomoxir for fatty acid oxidation (FAO) dependency and CB-839 for glutamine dependency), in purified NS and STIM B cells from WT and *Tfam* KO mice. Data were analysed using two-way ANOVA and are representative of 3 independent experiments

(C) Translation levels according to MFI of incorporated puromycin in purified B cell populations along GC maturation (FO, FO^{GL7}, pre-GC and mat-GC) in immunised WT and *Tfam* KO mice. Cells were incubated with vehicle (Ctrl), 2-Deoxi-D-glucose (2DG), Oligomycin (Olig), Etomoxir (Eto), and CB-839 (CB). A combination of all inhibitors (All) was used as a negative control for translation.

(D) Scheme of the cellular metabolic pathways. Enzymes used in the Met-flow panel are shown in red.

In panels A-C, bar charts show the quantification of one representative experiment out of three, and error bars represent mean +- SEM. Each dot represents one mouse. For panel A and C unpaired two-tailed t-test was conducted, and form panel B Two-way ANOVA was conducted with Tukey's multiple comparisons test: * p<0.05, ** p<0.01, ***p<0.001and ****p<0.0001. Source data are provided as a Source Data file.

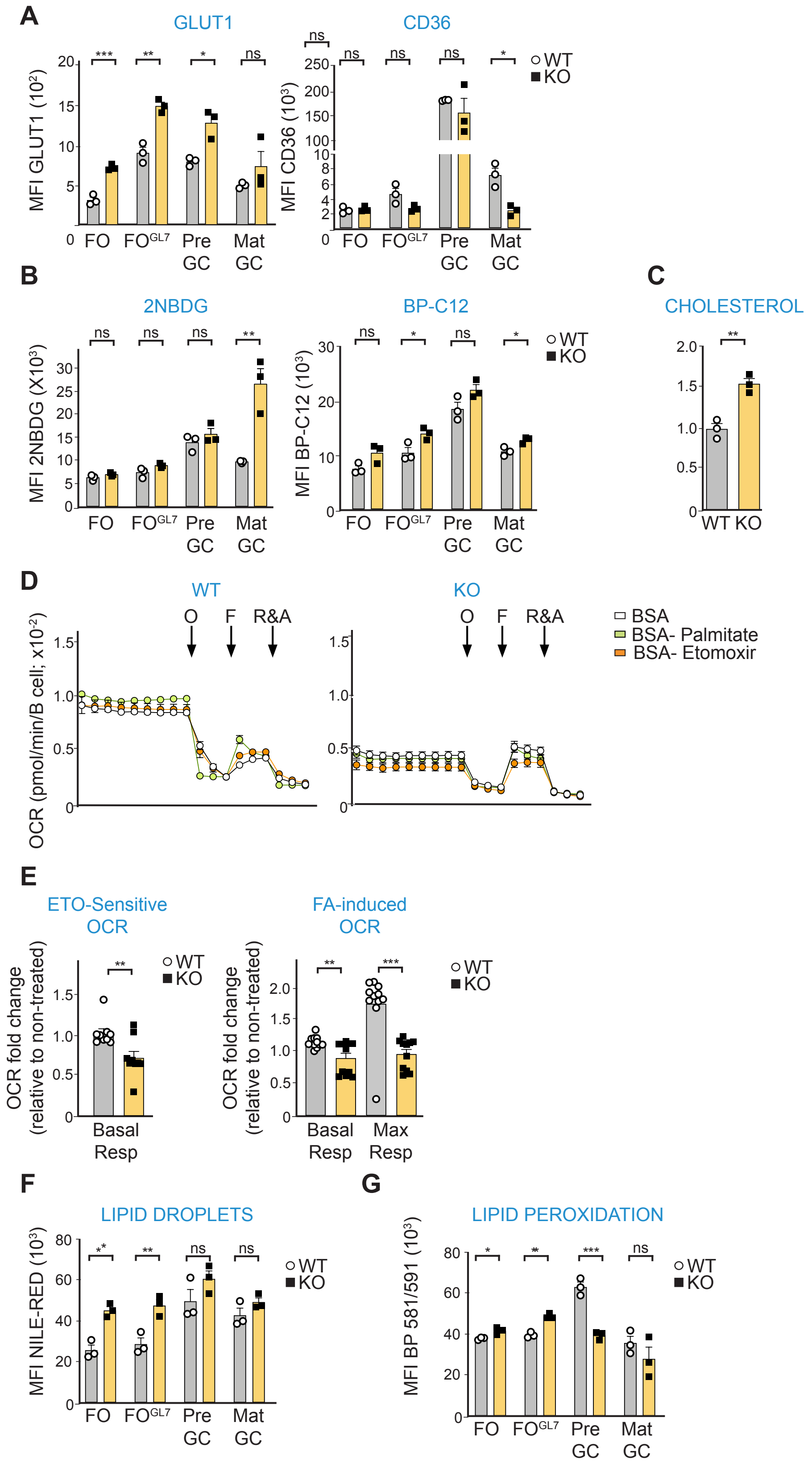
A
GLUT1
CD36
MFI GLUT1 (10²)
MFI CD36 (10³)
WT
KO
FO FO^GL7 Pre GC Mat GC
B
2NBDG
BP-C12
MFI 2NBDG (X10³)
MFI BP-C12 (10³)
C
CHOLESTEROL
WT KO
D
WT
KO
O F R&A
OCR (pmol/min/B cell; x10⁻²)
BSA
BSA- Palmitate
BSA- Etomoxir
E
ETO-Sensitive OCR
FA-induced OCR
OCR fold change (relative to non-treated)
Basal Resp
Max Resp
F
LIPID DROPLETS
MFI NILE-RED (10³)
G
LIPID PEROXIDATION
MFI BP 581/591 (10³)

Suppl. Fig. 14- *Tfam* deletion in B cells triggers metabolic plasticity to maintain bioenergetics homeostasis.

(A) Bar charts show levels of GLUT1 and CD36 along GC maturation (FO to mat-GC).

(B) Bar charts indicate glucose and fatty acid incorporation levels upon incubation with fluorescence 2NBDG and Bodipy FL C12, respectively, along GC maturation (FO to mat-GC).

(C) Bar charts indicating the relative amount of cholesterol in cell lysates of 24h-stimulated B cells from WT and *Tfam* KO mice.

(D) Representative OCR trace of 24h STM WT and *Tfam* KO B cells pretreated with Etomoxir (injected in port) used to identify the contribution of endogenous FA to oxygen consumption. Traces marked BSA-Palmitate, and BSA were from wells pretreated with palmitate-conjugated BSA, used to determine the contribution of exogenous FAs to oxygen consumption.

(E) Bar chart indicates normalized OCR values of WT and *Tfam* stimulated B cells relative to non-treated WT cells after injection of Etomoxir. Left, basal and maximal respiration rate in cells incubated with Palmitate-BSA or BSA.

(F-G) Bar charts indicate lipid content measured as lipid droplets (F; Nile Red) and FAO-derived oxidative stress (G; lipid peroxidation) in B cell populations along GC maturation (FO to mat-GC).

In panels A-C and E-G, bar charts show the quantification of one representative experiment out of three, and error bars represent mean +- SEM. Each dot represents one mouse. Unpaired two-tailed t-test: * $p<0.05$, ** $p<0.01$, ***$p<0.001$and ****$p<0.0001$. Source data are provided as a Source Data file.

Suppl Fig 15

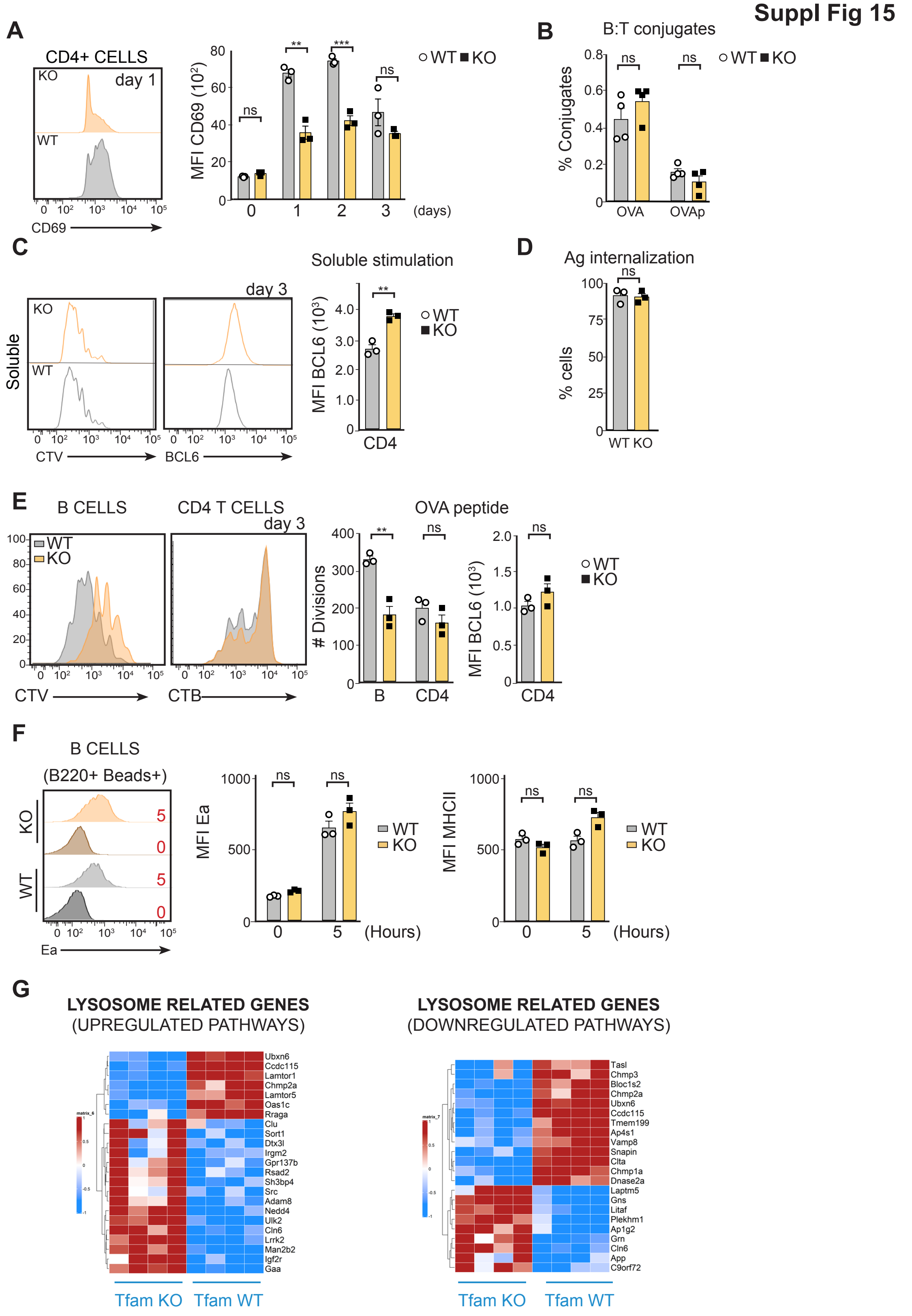

A
CD4+ CELLS
KO
day 1
WT
CD69
MFI CD69 (10²)
(days)
WT KO
B
B:T conjugates
% Conjugates
OVA
OVAp
C
Soluble stimulation
day 3
Soluble
CTV
BCL6
MFI BCL6 (10³)
CD4
D
Ag internalization
% cells
WT KO
E
B CELLS
CD4 T CELLS
OVA peptide
Divisions
CTB
F
B CELLS
(B220+ Beads+)
MFI Ea
MFI MHCII
(Hours)
Ea
G
LYSOSOME RELATED GENES
(UPREGULATED PATHWAYS)
LYSOSOME RELATED GENES
(DOWNREGULATED PATHWAYS)
Tfam KO
Tfam WT

Suppl. Fig. 15.-*Tfam* deletion in B cells impairs antigen presentation to CD4$^+$ cells.

(A) Representative flow cytometry histogram and quantification of CD69 levels in purified OT-II CD4$^+$ T cells co-cultured with WT and *Tfam* KO B cells.

(B) The bar chart illustrates the percentage of B-T conjugates (gated in live cells). B cells were incubated for 30 minutes with IgM along with either OVA protein or OVA peptide, followed by a 45-minute co-culture at 37ºC to facilitate cell interactions.

(C) Histograms of CTV dilution and Bcl6 protein levels in OT-II CD4$^+$ T cells co-cultured with purified WT and *Tfam* KO B cells. Cells were stimulated with soluble antigen (anti-IgM, anti-CD40, anti-CD3 and anti-CD28) for 3 days. Bar charts show Bcl6 levels quantification at day 3 post stimulation.

(D) Graph indicating the percentage of B cells with internalised antigen.

(E) Left, histograms of CTV and CTB of labelled B and OT-II CD4 T cells, respectively, after 3 days of co-culture, stimulated with OVA peptide 323-339 (OVAp). Right bar chart indicates the number of divisions calculated from cell trace dilutions in B and CD4 T cells and MFI levels of Bcl6 of CD4 T cells after 3 days of co-culture.

(F) On the left, surface expression levels of MHCII:Eα in B cells pre-incubated with Eα peptide and IgM-coated microspheres for 0 and 5 hours are shown. The middle section presents bar charts illustrating MHCII:Ea mean fluorescence intensity (MFI), while the right section displays total levels of MHCII.

(G) Heatmap represents the most differentially expressed genes (DEGs) between *Tfam* KO and WT B cells after 24h of stimulation with a padj value < 0.05 obtained by Benjamini–Hochberg correction. On the left side, the heatmap displays the scaled, normalized counts of DEGs associated with upregulated lysosome-related pathways in *Tfam* KO B cells. On the right side, the heatmap lists the scaled, normalized counts of DEGs associated with downregulated lysosome-related pathways in *Tfam* KO B cells

In panels A, B, C, D, E and F bar charts show the quantification of one representative experiment out of three, and error bars represent mean +- SEM. Each dot represents one mouse. Unpaired two-tailed t-test was conducted: **$p<0.01$ and ***$p<0.001$. Source data are provided as a Source Data file.

Suppl Fig 16

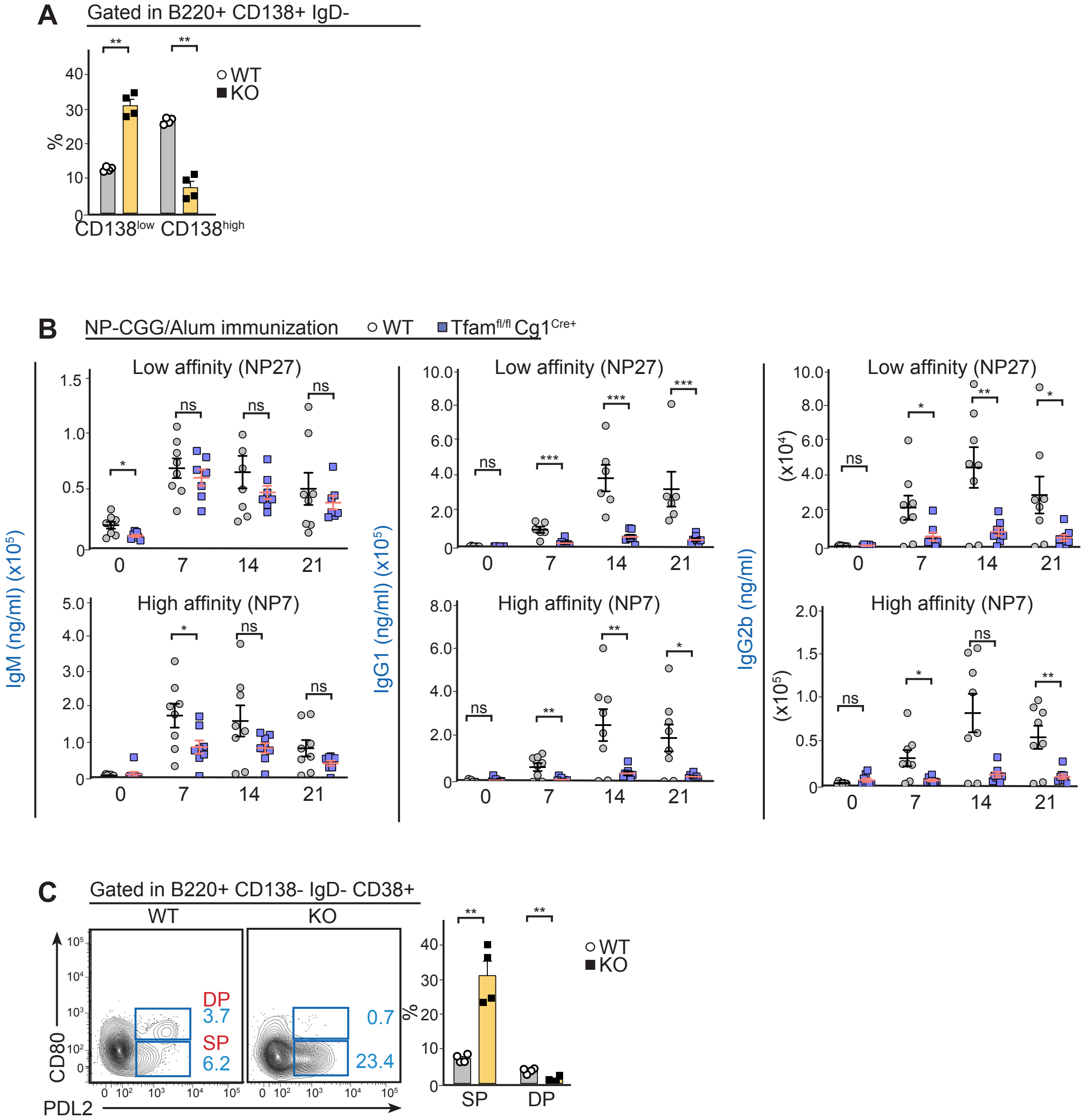

Suppl. Fig. 16.- *Tfam* deletion leads to defective immune response.

(A) Bar chart shows the percentage of antibody-secreting cell subsets: CD138^{low} (SLPC) and CD138^{high} (LLPC) in immunised WT and *Tfam* KO mice.

(B) NP-specific IgM, IgG1 and IgG2b titters on the specified days were determined by ELISA.

(C) Left, flow cytometry plots showing memory cell populations according to CD80 and PDL2 expression, referred as SP (PDL2^{+}, CD80^{-}) and DP (PDL2^{+}, CD80^{+}) in B cells gated in

splenocytes gated in $B220^+$, $CD138^-$, IgD^-, $CD38^+$. Right, the SP and DP population percentage in immunised WT and *Tfam* KO mice.

In all panels, bar charts show the quantification of one representative experiment out of three, and error bars represent mean +- SEM. Each dot represents one mouse. For panel A-C Two-way ANOVA was conducted with Tukey's multiple comparisons test; in panel B unpaired two-tailed t-test: * $p<0.05$, ** $p<0.01$, and ***$p<0.001$. Source data are provided as a Source Data file.

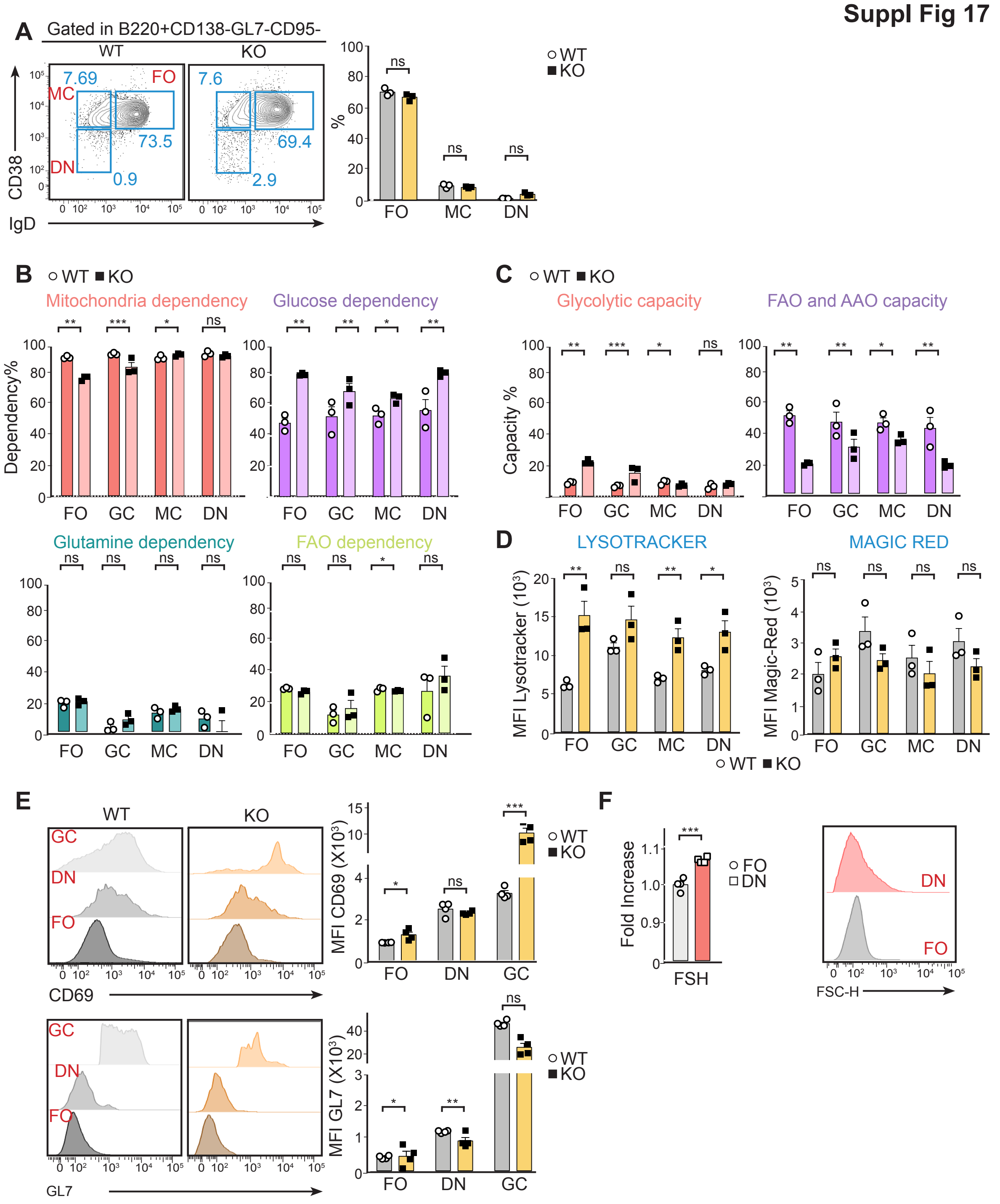

Suppl. Fig. 17.- Double Negative populations share similar features with GC cells.

(A) Flow cytometry plots showing the percentage of memory cells (MC), follicular cells (FO), and double negative cells (DN) based on levels of CD38 and IgD in non-immunised mice. Left, bar charts indicate the percentage of each cell type in WT and *Tfam* KO mice.

(B-C) Metabolic profile of Follicular (FO), total Germinal centre (GC), memory cell (MC) and double negative cell (DN) obtained by SCENITH as a result of incubation with different inhibitors B cells from WT and *Tfam* KO mice 7 days after immunisation.

(D) Lysosome content and activity were measured by Lysotracker and Magic Red. Bar charts indicating levels of lysotracker and magic red measured by FACS in GC, DN and FO cells in immunised WT and *Tfam* KO mice 7 days p.i

(E) Representative histograms of CD69 (upper panel) and GL7 (bottom panel) levels of GC, DN and FO cells in immunised WT and *Tfam* KO mice. Right, bar chart indicates MFI levels of CD69 and GL7.

(F) Left, quantification of the fold change increase of cell size of the double negative (DN) cell subset compared to the FO cell subset. Right representative histogram of FSC-H levels in DN and FO cells of immunised WT mice.

In all panels, bar charts show the quantification of one representative experiment out of three, and error bars represent mean +- SEM. Each dot represents one mouse. For panel A, two-way ANOVA was conducted with Tukey's multiple comparisons test. For panels B-F unpaired two-tailed t-test. P-values: * $p<0.05$, ** $p<0.01$, and ***$p<0.001$. Source data are provided as a Source Data file.

Suppl Fig 18

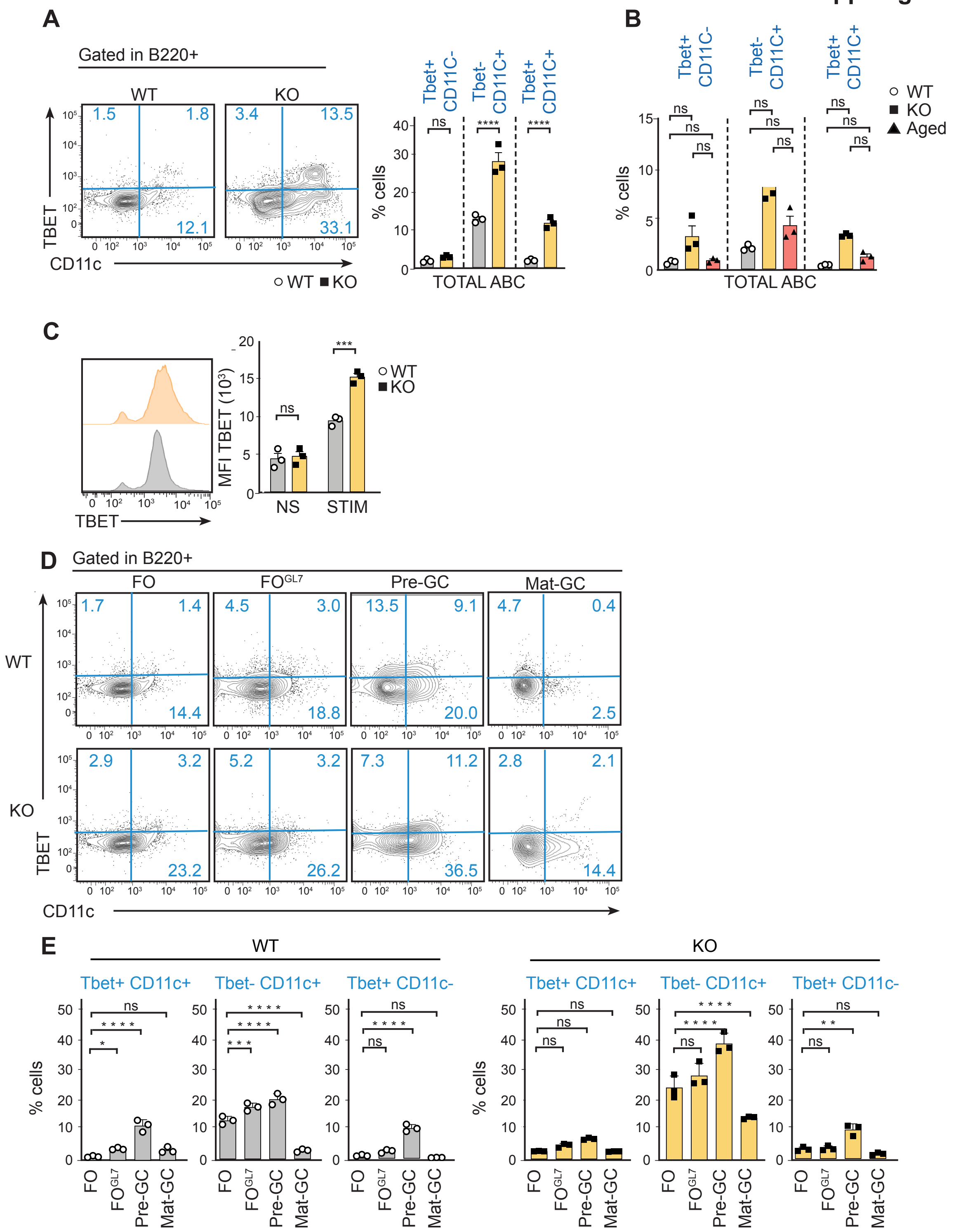
A
Gated in B220+
WT
KO
1.5
1.8
12.1
3.4
13.5
33.1
TBET
CD11c
○ WT ■ KO
Tbet+ CD11C-
Tbet- CD11C+
Tbet+ CD11C+
ns
****
****
% cells
TOTAL ABC
B
Tbet+ CD11C-
Tbet- CD11C+
Tbet+ CD11C+
○ WT
■ KO
▲ Aged
ns
% cells
TOTAL ABC
C
MFI TBET (10³)
***
ns
○ WT
■ KO
NS
STIM
TBET
D
Gated in B220+
FO
FOGL7
Pre-GC
Mat-GC
WT
KO
1.7
1.4
14.4
4.5
3.0
18.8
13.5
9.1
20.0
4.7
0.4
2.5
2.9
3.2
23.2
5.2
3.2
26.2
7.3
11.2
36.5
2.8
2.1
14.4
TBET
CD11c
E
WT
KO
Tbet+ CD11c+
Tbet- CD11c+
Tbet+ CD11c-
% cells
FO
FOGL7
Pre-GC
Mat-GC

Suppl. Fig. 18 *Tfam* deletion leads to an aged immune response.

(A-B) Total ABC quantification in B cells (gated in B220^{+}) from WT, *Tfam* KO (A) and WT-aged mice (>12 months) (B) 7 days after immunisation based on CD11c and Tbet distribution. Bar chart indicates the proportion of single-positive Tbet , single-positive CD11c and double-positive cells .

(C) Left, representative histogram Tbet levels in WT and *Tfam* KO stimulated 96hr in vitro with anti-CD40 and anti-IgM.

(D-E) Dot plot representation (D) of Tbet and CD11c distribution along GC maturation (FO to mat-GC) of immunised WT (upper panel) and *Tfam* KO (lower panel) mice. The bar chart (E) indicates the proportion of single-positive Tbet, single-positive CD11c, and double-positive cells.

In all panels, bar charts show the quantification of one representative experiment out of three, and error bars represent mean +- SEM. Each dot/ represents one mouse. For panel A, B, and E, two-way ANOVA was conducted with Tukey's multiple comparisons test; for panel C . unpaired two-tailed t-test: **P< 0,01, ***p<0.001 and ****p<0,0001. Source data are provided as a Source Data file.

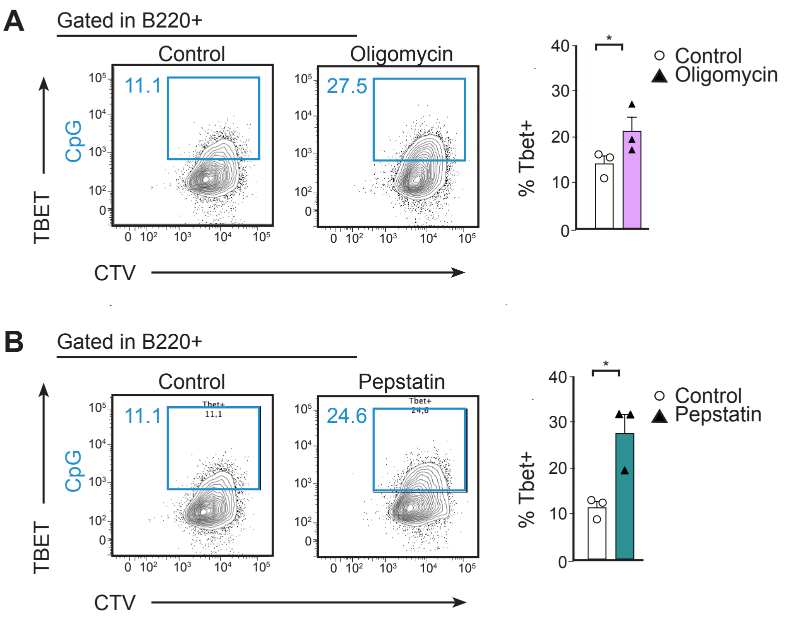

Suppl. Fig. 19 Targeting of mitochondrial and lysosome function leads to expansion of Tbet+ cells *in vitro*.

(A-B) Dot plot representation of Tbet and CTV distribution in isolated WT B cells (gated in B220+) culture for 3 days with IgM/CD40 and treated with Oligomycin (A) or pepstatin A (B). Right, bar chart indicates the percentage of Tbet+ cells in left-gated plots.

In all panels, bar charts show the quantification of one representative experiment out of three, and error bars represent mean +- SEM. Each dot represents one mouse. Unpaired two-tailed t-test: *p< 0,05. Source data are provided as a Source Data file.

Supplementary table 1

REAGENT or RESOURCE	SOURCE	IDENTIFIER
**Antibodies**		
Anti-CD16/32 Clone [93] dilution (1/100)	Biolegend	Cat #101330
Anti-B220 Clone [RA3-6B2] dilution (1/400)	Biolegend	Cat #103224
Anti-CD19 Clone [6D5] dilution (1/400)	Biolegend	Cat #115520
Anti-CD95 Clone [Jo2] dilution (1/200)	BD Biosciences	Cat No 557653
Anti-GL7 Clone [GL7] dilution (1/200)	Biolegend	Cat #144614
Anti-IgD Clone [11-26c.2a] dilution (1/400)	Biolegend	Cat #405742
Anti-IgM Clone [RMM-1] dilution (1/400)	Biolegend	Cat #406509
Anti-CD38 Clone [90] dilution (1/400)	BD Biosciences	Cat No 740887
Anti-CD138 Clone [281-2] dilution (1/300)	Biolegend	Cat #142516
Anti-CD69 Clone [H1.2F3] dilution (1/400)	Biolegend	Cat #164204
Anti-CD86 Clone [GL1] dilution (1/100)	Biolegend	Cat #105016
Anti-CXCR4 Clone [2B11] dilution (1/100)	Invitrogen	Cat #13-9991-82
Anti-CCR6 Clone [29-2L17] dilution (1/200)	Biolegend	Cat #129816
Anti-PDL2 Clone [TY25] dilution (1/200)	Biolegend	Cat #107210
Anti-Ckit (CD117) Clone [2B8] dilution (1/200)	Biolegend	Cat #105808
Anti-CD23 Clone [B3B4] dilution (1/200)	Biolegend	Cat #101606
Anti-CD21/CD35 Clone [7G6] dilution (1/200)	BD Biosciences	Cat No 740703
Anti-CD5 Clone [53-7.3] dilution (1/100)	BD Biosciences	Cat No 553019
Anti-CD11b Clone [M1/70] dilution (1/200)	Biolegend	Cat #101228
Anti-CD11c Clone [N418] dilution (1/200)	Biolegend	Cat #117308
Anti-CD4 Clone [RM4-4] dilution (1/400)	Biolegend	Cat #116027
Anti-2C11 (CD3) Clone [145-2C11] dilution (1/400)	Biolegend	Cat #100308
Anti-CD44 Clone [IM7] dilution (1/300)	Biolegend	Cat #103040
Anti-PD1 Clone [J43] dilution (1/100)	Invitrogen	Cat #12-9985-83
Anti-CXCR5 Clone [L138D7] dilution (1/100)	Biolegend	Cat #145510
Anti-CD98 Clone [RL388] dilution (1/400)	Biolegend	Cat #128214
Anti-CD36 Clone [HM36] dilution (1/100)	Biolegend	Cat #102606
Anti-Bcl6 Clone [K112-91] dilution (1/100)	BD Biosciences	Cat No 561522
Anti-Tbet Clone [4B10] dilution (1/100)	Biolegend	Cat #644814
Anti-Ki67 Clone [16A8] dilution (1/100)	Biolegend	Cat #652404
Anti-cleaved Caspase3 dilution (1/100)	Cell signalling	9661S
P-S6 Ribosomal Protein Ser240/244 dilution (1/100)	Cell signalling	5364S
Phospho-Akt (Thr308) dilution (1/100)	Cell Signalling	9275
Phospho-p44/42 MAPK (Erk1/2) (Thr202/Tyr204) dilution (1/100)	Cell Signalling	9101
Anti-G6PD Clone EPR20668 dilution (1/100)	Abcam	ab210702
Anti-Glut1 Clone EPR3915 dilution (1/100)	Abcam	ab210438
Anti-ASS1 Clone EPR12398 dilution (1/100)	Abcam	ab170952
Anti-PRDX2 Clone EPR5154 dilution (1/100)	Abcam	ab109367
Anti-ATP5A Clone EPR13030 (B) dilution (1/100)	Abcam	ab176569
Anti-ACAC Clone EPR23235-147 dilution (1/100)	Abcam	ab269273
Anti-Hexokinase 1 Clone EPR10134 (B) dilution (1/100)	Abcam	ab150423
Anti-CPT1A Clone 8F6AE9 dilution (1/100)	Abcam	ab128568
Anti-IDH2 Clone EPR7577 dilution (1/100)	Abcam	ab131263

Anti-Tfam	Abcam	ab252432
Anti-USP7 dilution (1/2000)	Bionova	A300-033A-M
Complex I anti-NDUFA9 (1/2000)	Abcam	ab14713
Complex II anti-SDHA Clone [2E3GC12FB2AE2] (1/2000)	Invitrogen	459200
Complex III anti-UQCR2 (1/2000)	Proteintech	14742-1-AP
Complex IV anti-CO1 Clone [1D6E1A8](1/2000)	Invitrogen	MTCO1 459600
Complex V (1/2000)	internal source	homemade anti-mouse IF-1
anti-vinculin	Sigma	Cat# AB6039
Anti-α-Tubulin Clone [DM1A]	Sigma	Cat# 05-829
Anti-LAMP1 clone [1D4B]	Sigma	Cat# MABC39
Anti-MHCII-Eα (1/500)	eBioscience	Cat #11-5741-82

Supplementary table 2

REAGENT or RESOURCE	SOURCE	IDENTIFIER
**Chemical, peptides and recombinant proteins**		
Streptavidin-APC dilution (1/400)	Biolegend	405207
anti-rabbit peroxidase	GE Healthcare	NA934
anti-mouse-HRP	Jackson immunoresearch	115-035-174
strep-HRP	Jackson immunoresearch	016-030-084
DyLight® 405 Conjugation kit	Abcam	Ab201798
DyLight® 405 Conjugation kit	Abcam	Ab201804
PE/CY7® Conjugation kit-Lightning-link	Abcam	Ab102859
PE/CY7® Conjugation kit-Lightning-link	Abcam	Ab10290
Alexa Fluor 647 anti-IgM	Jackson ImmunoResearch	115-605-075
Alexa Fluor 488 goat anti-mouse IgG1	Thermofisher	A-21121
Alexa Fluor 555 goat anti-rat IgGs	Thermofisher	A-31572
Mouse Immunoglobulin Panel	Southern Biotech	5300-01
SBA Clonotyping System-HRP	Southern Biotech	5300-05
CFSE CellTrace	ThermoFisher	C34554
Cell trace Violet	ThermoFisher	C34557
Cell trace Blue	ThermoFisher	C34568
Ghost-dye violet 540	TONBO Biosciences	13-0879
Ghost-dye red 780	TONBO Biosciences	13-0865
CpG ODN 1826 - TLR9 ligand	InvivoGen	tlrl-1826-1
PMA	Sigma	79346
Ionomycin	Sigma	IO-634
BrefeldinA	eBioscience	00-4506-51
anti-CD40 Clone [HM40-3]	Invitrogen	16-0402-85
anti-IgM F(ab)2	Jackson ImmunoResearch	115-006-075
anti-2C11	Internal source	
anti-CD28 Clone [37.51]	BioXCell	Cat #BE0015-1
Anti-IgM biotinylated Clone [II/41]	BD Biosciences	Cat# 553436
Interleukin-4	Peprotech	214-14
Interleukin-5	Peprotech	214-15
Interleukin-21	Peprotech	210021
IFNg	Peprotech	315-05
Etomoxir	Sigma	E1905-5MG
CB-839	Sigma	5. 33717
Oligomycin A	Sigma	75351
Rotenone	Sigma	R8875
Antimycin A	Sigma	A8674
PepstatinA	Sigma	P5318
MitoTRacker Green	ThermoFisher	M7514
MitoTracker Red CMXROS	ThermoFisher	M7512
CM-H2DCFDA	ThermoFisher	C6827
QUINACRINE	Sigma	Q3251-25G
Bodipy™ C12	ThermoFisher	D3822
BODIPY™ 581/591	ThermoFisher	D2228

Nile Red	Sigma	Cat# N3013
2-NBDG	Abcam	ab235976
Lysotracker Green DND-26	Thermofisher	L7526
Magic RED ® Cathepsin-B Assay Kit	Immunochemestry Technologies	Catalog #937
Albumin from chicken egg white	Sigma	A5503
B cell isolation kit, mouse	Miltenyi Biotec	130-090-862
CD4+ T Cell Isolation Kit, mouse	Miltenyi Biotec	130-104-454
Seahorse XF Cell Mito Stress Test Kit	Agilent	103010-100
Seahorse XF Real-Time ATP Rate Assay Kit	Agilent	103591-100
Seahorse XF Palmitate-BSA FAO Substrate	Agilent	102720-100
SCENITH	Internal source CNRS	Ref (60)

Supplementary table 3

REAGENT or RESOURCE	SOURCE
**Oligonucleotides**	
Tfam FW CAGGAGGCAAAGGATGATTC (2,5μM)	IDT
Tfam RV CCAAGACTTCATTTCATTGTCG (2,5μM)	IDT
Mt-nd1 FW TCCCCTACCAATACCACACC (2,5μM)	IDT
Mt-nd1 RV TGAAATTGTTTGGGCTACGG (2,5μM)	IDT
Mt-co1 FW ATCCCTTGACATCGTGCTTC (2,5μM)	IDT
Mt-co1 RV AAGTGGGCTTTTGCTCATGT (2,5μM)	IDT
Mt-atp6 FW ACAGGCTTCCGACACAAACT (2,5μM)	IDT
Mt-atp6 RV GGTAGCTGTTGGTGGGCTAA (2,5μM)	IDT
Sdha FW GCGGCTTTCACTTCTCTGTT (2,5μM)	IDT
Sdha RV CTCAGAAAGGCCAAATGCAG (2,5μM)	IDT
