# Supplementary material for: Defective mitochondria remodelling in B cells leads to an aged immune response
Source: Nat Commun. 2024 Mar 22;15:2569. doi: 10.1038/s41467-024-46763-1 (PMC10960012; doi:10.1038/s41467-024-46763-1)
Supplement: Supplementary file 3 — Description of Additional Supplementary Files [file 41467_2024_46763_MOESM3_ESM.pdf]

Suppl. Data 1.- Comprehensive RNA-seq Data Analysis of *Tfam* KO vs WT B cells at 24 hours post-stimulation.

The table contains differential gene expression data of *Tfam* KO vs WT B cells, including log2 fold change values, p-values, and adjusted p-values. Additionally, the table provides normalized counts, raw counts, and Fragments Per Kilobase of transcript per Million mapped reads (FPKM) for each gene, along with gene information for each gene ID
