# Supplementary material for: Defective mitochondria remodelling in B cells leads to an aged immune response
Source: Nat Commun. 2024 Mar 22;15:2569. doi: 10.1038/s41467-024-46763-1 (PMC10960012; doi:10.1038/s41467-024-46763-1)
Supplement: Supplementary file 5 — Reporting Summary [file 41467_2024_46763_MOESM5_ESM.pdf]

nature portfolio

Corresponding author(s): Nuria Martínez-Martín

Last updated by author(s): 2023/12/08

Reporting Summary

Nature Portfolio wishes to improve the reproducibility of the work that we publish. This form provides structure for consistency and transparency in reporting. For further information on Nature Portfolio policies, see our Editorial Policies and the Editorial Policy Checklist.

Statistics

For all statistical analyses, confirm that the following items are present in the figure legend, table legend, main text, or Methods section.

n/a	Confirmed	
☐	☒	The exact sample size (*n*) for each experimental group/condition, given as a discrete number and unit of measurement
☐	☒	A statement on whether measurements were taken from distinct samples or whether the same sample was measured repeatedly
☐	☒	The statistical test(s) used AND whether they are one- or two-sided<br>*Only common tests should be described solely by name; describe more complex techniques in the Methods section.*
☒	☐	A description of all covariates tested
☒	☐	A description of any assumptions or corrections, such as tests of normality and adjustment for multiple comparisons
☐	☒	A full description of the statistical parameters including central tendency (e.g. means) or other basic estimates (e.g. regression coefficient) AND variation (e.g. standard deviation) or associated estimates of uncertainty (e.g. confidence intervals)
☐	☒	For null hypothesis testing, the test statistic (e.g. *F*, *t*, *r*) with confidence intervals, effect sizes, degrees of freedom and *P* value noted<br>*Give P values as exact values whenever suitable.*
☐	☒	For Bayesian analysis, information on the choice of priors and Markov chain Monte Carlo settings
☒	☐	For hierarchical and complex designs, identification of the appropriate level for tests and full reporting of outcomes
☒	☐	Estimates of effect sizes (e.g. Cohen's *d*, Pearson's *r*), indicating how they were calculated

Our web collection on statistics for biologists contains articles on many of the points above.

Software and code

Policy information about availability of computer code

Data collection	FACSDiva, SpectroFlo, Seahorse XFe96 Software package, ZEN, Bio-Rad CFX Maestro, Microplate Manager 6
Data analysis	RStudio (v 4.2.2), Graphpad Prism (v 9.5.1), Fiji (ImageJ), FlowJo (v 10.8.1), OMIQ, Bio-Rad CFX Maestro, Seahorse Analytics (v 1.0.0-684)

For manuscripts utilizing custom algorithms or software that are central to the research but not yet described in published literature, software must be made available to editors and reviewers. We strongly encourage code deposition in a community repository (e.g. GitHub). See the Nature Portfolio guidelines for submitting code & software for further information.

Data

Policy information about availability of data

All manuscripts must include a data availability statement. This statement should provide the following information, where applicable:

- Accession codes, unique identifiers, or web links for publicly available datasets
- A description of any restrictions on data availability
- For clinical datasets or third party data, please ensure that the statement adheres to our policy

The bulk cell RNA sequencing data generated in this study have been deposited in the Gene Expression Omnibus (GEO) data base under the accession number GSE249893 (https://www.ncbi.nlm.nih.gov/geo/query/acc.cgi?acc=GSE249893). Metabolomic data generated in this stady have been deposited in the Metabolomics Workbench: An international repository for metabolomics data and metadata, metabolite standards, protocols, tutorials and training, and analysis tools (2016)'. [PubMed: https://www.ncbi.nlm.nih.gov/pubmed/26467476/]. This study is available at the NIH Common Fund's National Metabolomics Data Repository (NMDR) website, the Metabolomics Workbench, https://www.metabolomicsworkbench.org where it has been assigned Study ID ST003047. The data can

be accessed directly via its Project DOI: http://dx.doi.org/10.21228/M8HH8R. This work is supported by NIH grant U2C-DK119886. The study is available for review at http://dev.metabolomicsworkbench.org:22222/data/DRCCMetadata.php?Mode=Study&StudyID=ST003047&Access=ThgJ8001. Source data are provided with this paper. Any additional information required to reanalyse the data reported in this paper is available from the lead contact upon request.

Research involving human participants, their data, or biological material

Policy information about studies with human participants or human data. See also policy information about sex, gender (identity/presentation), and sexual orientation and race, ethnicity and racism.

Reporting on sex and gender	N/A
Reporting on race, ethnicity, or other socially relevant groupings	N/A
Population characteristics	N/A
Recruitment	N/A
Ethics oversight	N/A

Note that full information on the approval of the study protocol must also be provided in the manuscript.

Field-specific reporting

Please select the one below that is the best fit for your research. If you are not sure, read the appropriate sections before making your selection.

[x] Life sciences [ ] Behavioural & social sciences [ ] Ecological, evolutionary & environmental sciences

For a reference copy of the document with all sections, see nature.com/documents/nr-reporting-summary-flat.pdf

Life sciences study design

All studies must disclose on these points even when the disclosure is negative.

Sample size	No statistical methods were used to pre-determine sample sizes. For all in vivo and in vitro experiments, minimum 3 biological replicates and minimum 2 independent experiments were performed. Samples size and number of independent experiments are indicated in the figure legends. Group sizes were chosen based on the typical number of replicates reported for similar studies as outlined in Festing MFW,"On determining sample size in experiments involving laboratory animals",2018 Aug;52 (4):341-350.doi:10.1177/0023677217738268,relyingongroupsizesofGreenetal.,FrontImmunol.2021Dec6;12:782558.doi:10.3389/fimmu.2021.782558; Voss et al.,Front Immunol.2022 Dec14;13:1021370.doi:10.3389/fimmu.2022.1021370;and Degn et al.,Cell.2017 Aug24;170(5):913-926.e19.doi:10.1016/j.cell.2017.07.026;for similar experimental set ups and read-outs.
Data exclusions	No data were excluded from analysis.
Replication	All experiments were repeated (> 2 times) and reproducible. RNA-Seq was not repeated. Conducting RNA sequencing (RNAseq) experiments demands significant resources in terms of time, cost, and sample availability. Limited resources hinder the feasibility of conducting multiple independent experiments. The chosen sample size of 4 replicates is deemed sufficient to achieve adequate statistical power, either through preliminary power analyses or through existing knowledge regarding the anticipated effect size and variance
Randomization	Randomization was not relevant to this study. Mice were grouped by genotype/treatment and treated equally.
Blinding	No blinding was performed as mice were grouped by genotype/and treated equally. Noblindingwasperformed.Sampleidentitywasinherentlyrepresentedbytheread-outs,precludingblindingduringgating.However, universalgatingwasemployedforeachexperiment.Allread-outswerequantitative,ratherthanqualitative,minimizingtheriskof experimenterbiasininterpretingtheresults.

Reporting for specific materials, systems and methods

We require information from authors about some types of materials, experimental systems and methods used in many studies. Here, indicate whether each material, system or method listed is relevant to your study. If you are not sure if a list item applies to your research, read the appropriate section before selecting a response.

Materials & experimental systems

n/a	Involved in the study	
[ ]	[x]	Antibodies
[x]	[ ]	Eukaryotic cell lines
[x]	[ ]	Palaeontology and archaeology
[ ]	[x]	Animals and other organisms
[x]	[ ]	Clinical data
[x]	[ ]	Dual use research of concern
[x]	[ ]	Plants

Methods

n/a	Involved in the study	
[x]	[ ]	ChIP-seq
[ ]	[x]	Flow cytometry
[x]	[ ]	MRI-based neuroimaging

Antibodies

Antibodies used

Antibodies used in this study are detailed in Material & Methods.

Validation

BioLegend
https://www.biolegend.com/en-us/quality/quality-control
Specificity testing of 1-3 target cell types with either single- or multi-color analysis (including positive and negative cell types).
Once specificity is confirmed, each new lot must perform with similar intensity to the in-date reference lot. Brightness (MFI) is evaluated from both positive and negative populations.
Each lot product is validated by QC testing with a series of titration dilutions.

BDBiosciences
https://www.bdbiosciences.com/en-eu/products/reagents/flow-cytometry-reagents/research-reagents/quality-and-reproducibility
BD Biosciences not only develops its own antibodies but also collaborates with research scientists around the world to license their antibodies.
We provide accessibility to the flow cytometry community by conjugating antibodies to a broad portfolio of high-performing dyes, including our vastly popular portfolio of BD Horizon Brilliant™ Dyes.
A world-class team of research scientists helps ensure that these reagents work reliably and consistently for flow cytometry applications.
The specificity is confirmed by using multiple applications that may include a combination of flow cytometry, immunofluorescence, immunohistochemistry or western blot to test a combination of primary cells, cell lines or transfectant models.
All flow cytometry reagents are titrated on the relevant positive or negative cells. To save time and cell samples for researchers, pre-titrated test size reagents are bottled at an optimal concentration, with the best signal-to-noise ratio on relevant models. You can look up the Certificate of Analysis and the concentration of test-size human reagents from specific lots via the Concentration Lookup page or BD Regulatory Documents.
Technical data sheets provide data generated on the relevant primary model at this optimal concentration based on a titration curve. QC data on any lot of reagent can be requested through ResearchApplications@bd.com.
Quality control testing of new, manufactured lots are performed side-by-side with a previously accepted lot as a control, helping to serve as a reference for comparison and assuring that performance of the new lot is both reliable and consistent.
Our strict adherence to these guidelines helps ensure that different lots of conjugated reagents are performing consistently.
We strive to ensure different production batches are consistent regardless of type of antigen or fluorochrome through exhaustive testing and strict adherence to quality control standards. Testing with prior batches as reference helps you obtain consistent results with the new batch relative to the previous batches.

eBioscience/Thermofisher/Invitrogen
https://www.thermofisher.com/es/es/home/life-science/antibodies/antibody-performance-guarantee.html
Each antibody is manufactured with high quality to enable customers to obtain reproducible results.
Each antibody is designed to meet the applicable specification as stated on the product data sheets, including but not limited to species reactivity, application suitability, and detection of target of interest as identified by the stated UniProt ID.

Miltenyi Biotec
https://www.miltenyibiotec.com/ES-en/products/macs-antibodies/antibody-validation.html
All our antibodies are rigorously tested and validated before release. In the application section on the product page, you can find examples of typical performance data. In addition, we provide extended validation data highlighting details of antibody performance, specificity, and fixation compatibility. All antibodies for which any of these datasets are already available will be indicated with the extended validation stamp.

Cell Signalling
https://www.cellsignal.com/about-us/cst-antibody-validation-principles
At Cell Signaling Technology (CST), we understand that there is no single assay that can determine the validity of an antibody. Confirming that an immunoreagent is sufficiently specific and sensitive depends on the application and protocol being used, the type and quality of sample being analyzed, and the inherent biophysical properties of the antibody itself.
To ensure our antibodies will work in your experiment, we adhere to the Hallmarks of Antibody Validation™, six complementary strategies that can be used to determine the functionality, specificity, and sensitivity of an antibody in any given assay. CST adapted the work by Uhlen, et. al., ("A Proposal for Validation of Antibodies." Nature Methods (2016)) to build the Hallmarks of Antibody Validation, based on our decades of experience as an antibody manufacturer and our dedication to reproducible science.
We guarantee that our antibodies are fit for purpose by carefully tailoring the combination of validation strategies applied to each

product. This means customizing our validation process according to the biological role of the target, while considering the sensitivity requirements of the downstream assay, the availability of appropriate testing models, and the relevance of each method to target investigation.

Abcam
https://www.abcam.com/primary-antibodies/highly-validated-antibody-conjugates
Highly validated: Whenever possible, we take extra steps to validate our primary conjugates through ICC/IF testing to confirm the signal localization. Thorough validation means you can have greater confidence in your results from the very start of your experiments.
Consistent batches: Recombinant manufacture offers the highest batch-to-batch consistency – paired with stringent quality checks, you can be confident of reproducible results with reduced variability.
Ensured specificity: Our conjugated recombinant primary antibodies directly bind to the target of interest, bringing specificity and accuracy to your results. Our quality assurance process is designed to reduce variability and ensure a high signal-to-noise ratio during your experiments involving the engineering of recombinant fluorescent antibody conjugates or removing free dye.
https://www.abcam.com/nav/primary-antibodies
We are committed to addressing the reproducibility crisis in research, so knock-out (KO) validation is always part of our processes where possible, and we have recently introduced biophysical testing during quality control stages. We extensively validate across applications including flow cytometry, western blot, immunohistochemistry (IHC), and immunocytochemistry (ICC/IF). We review our validation data 80,000 times per year.

GE Healthcare
https://gels.yilimart.com/Assets/Images/doc/file/NA934-1ML_INSTRUCTION_09.PDF
For every batch of enzyme-linked antibody that is produced the antibody titre is determined in an ELISA. The substrate used for the peroxidase is 2,2'-Azinobis[3-Ethylbenzothiazoline Sulphonate, diammonium salt], ABTS™.
Every batch is also QC tested in a Western blotting system. This is performed using Hybond™ ECL™ membrane containing serially diluted Beta-galactosidase protein and immundetected with primary antibody Anti-Beta-galactosidase and secondary antibody NA934, anti-rabbit HRP. Blots are detected using ECL and ECL Plus™ detection systems.

Jackson Immunoresearch
https://www.jacksonimmuno.com/company/iso
The quality management systems of Jackson ImmunoResearch Europe Ltd. and our parent company Jackson ImmunoResearch Laboratories Inc. are registered under ISO 9001:2015 as part of our ongoing commitment to provide quality manufacturing.
Jackson ImmunoResearch Europe Ltd is certified by NQA for the operations of stockholding and distribution of life science research consumables, including provision of technical support. View Jackson ImmunoResearch Europe Ltd. ISO 9001:2015 quality certificate.

Sigma
https://www.sigmaaldrich.com/ES/en/products/protein-biology/antibodies
Our highly cited primary, secondary, and recombinant monoclonal antibodies have you covered for your ELISA, Western blot, immunohistochemistry, or other assay needs. We are proud to offer brands you know and trust, including Sigma-Aldrich® antibodies, Millipore® antibodies, Upstate antibodies, Chemicon antibodies, and Calbiochem antibodies. ZooMAb® recombinant monoclonal antibodies provide high reproducibility and are validated across at least three applications, hybridoma-free, preservative-free, and produced animal-free.

DSHB
https://dshb.biology.uiowa.edu/1D4B

Southern Biotech
https://www.southernbiotech.com/antibody-characterization-and-testing/
SouthernBiotech offers antibody characterization services for any antibody, whether made by us or made elsewhere and shipped to us for testing. We routinely use a variety of techniques to evaluate the purity and performance of the antibodies we sell, and extend that proven expertise to all antibody characterization and testing service projects.

Animals and other research organisms

Policy information about studies involving animals; ARRIVE guidelines recommended for reporting animal research, and Sex and Gender in Research

Laboratory animals	Tfamfl/fl mice were provided by Larsson NG and were crossed at the Centro de Biología Molecular Severo Ochoa (CBM) animal facility with MB1Cre mice. MB1Cre and OT2 mice were kindly provided by Alarcón B. All mice were bred and maintained under specific pathogen-free conditions at the CBM Severo Ochoa animal facility, following national and European guidelines.<br><br>Strain: C57BL/6. Male and female between the ages of 8-16 weeks were used for all experiments.
Wild animals	No wild animals were used in this study
Reporting on sex	Both male and female mice between 8 and 24 weeks of age at the time of the experiment were used for this study. Mice >12 months of age were used as "aged" mice.
Field-collected samples	No field-collected samples were used in this study.

Ethics oversight	The ethical committee of the CBM Severo Ochoa approved all procedures.

Note that full information on the approval of the study protocol must also be provided in the manuscript.

Plants

Seed stocks	N/A
Novel plant genotypes	N/A
Authentication	N/A

Flow Cytometry

Plots

Confirm that:

- [x] The axis labels state the marker and fluorochrome used (e.g. CD4-FITC).
- [x] The axis scales are clearly visible. Include numbers along axes only for bottom left plot of group (a 'group' is an analysis of identical markers).
- [x] All plots are contour plots with outliers or pseudocolor plots.
- [x] A numerical value for number of cells or percentage (with statistics) is provided.

Methodology

Sample preparation	A single cell suspension was prepared from the spleen and lymph nodes of donor mice. Red blood cell were lysed by lysis buffer before samples were used for B or CD4+ cell isolation or FACS analysis.
Instrument	Data was collected on a BD FACSCantoII or Cytek Aurora. Cell sorting was performed in a BD FACSAria Fusion.
Software	BD FlowJo (v 10.8.1) and OMIQ was used for analysis.
Cell population abundance	Post Sort analysis was routinely performed on sorted samples and the target population was above 90%total eventsand about 95% of cells in a FSC-A/SSC-A lymphocyte gate.
Gating strategy	Dead cells were excluded by viability staining, and singlets were gated by FSC-A/FSC-H. Gating strategy is explained within the text.

- [ ] Tick this box to confirm that a figure exemplifying the gating strategy is provided in the Supplementary Information.
